# Effect of genotyping strategies on the sustained benefit of single-step genomic BLUP over multiple generations

**DOI:** 10.1186/s12711-022-00712-y

**Published:** 2022-03-18

**Authors:** Milagros Sánchez-Mayor, Valentina Riggio, Pau Navarro, Beatriz Gutiérrez-Gil, Chris S. Haley, Luis Fernando De la Fuente, Juan-José Arranz, Ricardo Pong-Wong

**Affiliations:** 1 Dpto. Producción Animal, Universidad de León, 24071 León, Spain; 2 The Roslin Institute and R(D)SVS, University of Edinburgh, Easter Bush Campus, Edinburgh, EH25 9RG UK; 3 Centre for Tropical Livestock Genetics and Health (CTLGH), Roslin Institute, University of Edinburgh, Easter Bush Campus, Edinburgh, EH25 9RG UK; 4 MRC Human Genetics Unit, Institute of Genetics and Cancer, University of Edinburgh, Western General Hospital, Crewe Road, Edinburgh, EH4 2XU UK

## Abstract

**Background:**

Single-step genomic best linear unbiased prediction (ssGBLUP) allows the inclusion of information from genotyped and ungenotyped individuals in a single analysis. This avoids the need to genotype all candidates with the potential benefit of reducing overall costs. The aim of this study was to assess the effect of genotyping strategies, the proportion of genotyped candidates and the genotyping criterion to rank candidates to be genotyped, when using ssGBLUP evaluation. A simulation study was carried out assuming selection over several discrete generations where a proportion of the candidates were genotyped and evaluation was done using ssGBLUP. The scenarios compared were: (i) three genotyping strategies defined by their protocol for choosing candidates to be genotyped (RANDOM: candidates were chosen at random; TOP: candidates with the best genotyping criterion were genotyped; and EXTREME: candidates with the best and worse criterion were genotyped); (ii) eight proportions of genotyped candidates (*p*); and (iii) two genotyping criteria to rank candidates to be genotyped (candidates’ own phenotype or estimated breeding values). The criteria of the comparison were the cumulated gain and reliability of the genomic estimated breeding values (GEBV).

**Results:**

The genotyping strategy with the greatest cumulated gain was TOP followed by RANDOM, with EXTREME behaving as RANDOM at low *p* and as TOP with high *p*. However, the reliability of GEBV was higher with RANDOM than with TOP. This disparity between the trend of the gain and the reliability is due to the TOP scheme genotyping the candidates with the greater chances of being selected. The extra gain obtained with TOP increases when the accuracy of the selection criterion to rank candidates to be genotyped increases.

**Conclusions:**

The best strategy to maximise genetic gain when only a proportion of the candidates are to be genotyped is TOP, since it prioritises the genotyping of candidates which are more likely to be selected. However, the strategy with the greatest GEBV reliability does not achieve the largest gain, thus reliability cannot be considered as an absolute and sufficient criterion for determining the scheme which maximises genetic gain.

**Supplementary Information:**

The online version contains supplementary material available at 10.1186/s12711-022-00712-y.

## Background

Genomic prediction (or genomic selection, GS) uses information that is derived from high-throughput genotyping of single nucleotide polymorphisms (SNPs) in the genetic evaluation [1]. It has been successfully implemented in many commercial breeding populations from several livestock species and has significantly increased the accuracy/reliability of the estimated breeding values (e.g. [2–8]). Other advantages that have been observed with GS are an increase in the intensity of the selection and shortening of the generation interval [9, 10].

Several methods that are defined by their assumption on the prior distribution of the SNP effects have been proposed and implemented (for a review of methods see Gianola et al. [11] and Gianola [12]), with the most popular ones being genomic best linear unbiased prediction (GBLUP) [13], and especially its subsequent development, known as single-step GBLUP or ssGBLUP [14–16]. The attractiveness of ssGBLUP is due to the fact that it allows to include information from genotyped and ungenotyped animals in a single joint analysis leading to better predictions in the genotyped animals and, to some extent, it propagates into the group of ungenotyped ones [14, 17].

Hence, an additional appeal of ssGBLUP is that it allows the option of not genotyping the whole group of candidate animals, which thereby reduces the cost of the selection scheme. However, this raises the need to determine the optimum set of candidates to be genotyped to ensure that a significant benefit from ssGBLUP is still achieved. In this study, the aim was to assess the effect of genotyping strategies, the proportion of genotyped candidates and the genotyping criterion to rank candidates to be genotyped on the cumulated genetic gain, when using ssGBLUP evaluation.

## Methods

A simulation study was performed to assess the effect of genotyping strategies on the response to selection over multiple generations using ssGBLUP to account for not all candidates being genotyped. A total of 100 replicates were used.

### Founder population in linkage disequilibrium

To simulate a genome in linkage disequilibrium (LD), a founder population was simulated using a mutation–drift-equilibrium algorithm as suggested by Meuwissen et al. [1]. Briefly, an initial population of $n$ individuals is allowed to reproduce, with each individual producing two offspring (one male and one female). The genome is assumed to be divided into several chromosomes with biallelic loci that mutate at a given rate. As the population develops over a large number of generations, new mutations appear and they are lost or they increase in frequency due to drift, resulting in a population with a genome with segregating linked loci in LD. Thereafter, the population is expanded to produce the founder population, which is used to sample the haplotype of the base animals of the breeding population. In order to simulate the genome with a similar LD pattern as a typical farmed sheep structure, we assumed an initial population of 100 individuals, which was allowed to reproduce for 10,000 generations. The genome was composed of 26 chromosomes of one Morgan. Each chromosome had 500,000 biallelic loci located equidistantly and a mutation rate of 10^–6^. Thereafter, the population had three generations of fivefold expansion and 4000 individuals of the last generation were randomly selected to be the founder population. This founder population was later used to sample the genome of the base population of each replicate (i.e. the G(− 2) generation, see below). In the founder population, approximately 5000 loci per chromosome were segregating at a minor allele frequency (MAF) higher than 0.05 (i.e. more than 130,000 loci across the whole genome).

### Population structure

The breeding population was assumed to have discrete generations, with 900 individuals (300 males and 600 females) per generation. At each generation, 30 males and 300 females were selected and mated, with each selected female having three offspring (1 male and 2 females). All individuals were assumed to have one phenotypic record available for their genetic evaluation. The aim of the simulated population structure was to mimic a sheep breeding population with large paternal and small maternal families. The assumption of a litter size of 3 can be considered as a little unrealistic in a sheep population, but it was the minimum possible size in order to assume discrete generations while still being able to have selection pressure on both male and female candidates. However, the assumption of discrete generations means that the results would not be affected by differences in generation interval, and the observed differences between scenarios would be due only to the quality of the estimates. Hence, the decision of assuming discrete generations.

The first two generations (G(− 2), G(− 1)) were assumed to be the initial reference population, so all individuals were genotyped. Thereafter, nine additional generations were simulated (G0 to G8), with only a proportion $p$ of the individuals being genotyped. The selection scheme using ssGBLUP started at G0, therefore the comparison between strategies was done from G0 to G8. For the selection period using the ssGBLUP, the candidates available for selection were a mixture of genotyped and ungenotyped individuals. All candidates were assumed to be phenotyped early in life so phenotypic performances were available at the time of their genetic evaluation and when the decision of which candidates to genotype was taken.

### Genetic architecture

The genome was divided into 26 autosomal chromosomes (i.e., mimicking the sheep genome), each with 1000 loci used as the SNP panel and 100 loci used as the quantitative trait loci (QTL) affecting the trait. The genome of the animals in the base population (i.e., G(− 2)) was simulated by randomly sampling haplotypes from the founder population, and in the following generations (i.e. G(− 1) to G8), an animal’s genome was sampled assuming Mendelian inheritance given its parents’ haplotype.

To select the loci included in the SNP panel and the QTL, we calculated the frequency of all segregating loci at G0 and, within each chromosome, we selected 1100 with the highest MAF. The distribution of MAF at G0 for all loci selected across replicates is shown in Additional file 1: Fig. S1. Thereafter, 1000 loci were randomly assigned to be part of the SNP panel and 100 to be the QTL (i.e., across the genome, the SNP panel had 26,000 loci and 2600 loci were QTL affecting the traits). This protocol means that the sets of loci used as QTL or as part of the SNP panel (used in the ssGBLUP evaluation) were different in each replicate.

The heritability of the trait was assumed to be 0.2 (i.e. the genetic variance was 20 and the environmental variance was 80). The additive effect for each QTL was sampled from a standardised normal distribution and the favourable allele was assigned randomly with equal probability. The true breeding value (TBV) of each individual was calculated as the sum of the QTL effects, given its genotypes and the additive effect. The QTL effects were rescaled, such that the genetic variance in G(− 2) was 20. The phenotype for each individual was simulated as the sum of its TBV and an environmental effect sampled from a normal distribution with a mean of zero and a variance of 80.

Simulating the genome of the base population (G(− 2)) by sampling from the permuted haplotypes of the expanded founder population ensured that all the replicates had the same expected LD pattern, but still allowing for the replicates to be independent from each other. The size of the founder population (in terms of number of animals and loci) was sufficiently large so that the permutation of the founders’ haplotypes resulted in all base animals having a unique genome structure (i.e. no pair of base animals, within or across replicates, had the same haplotype pattern). Furthermore, the loci assigned as SNPs or QTL (and their effects) were sampled within each replicate, further ensuring that replicates were different, and thereby, independent.

### Genetic evaluation and selection

The genetic evaluation was done using ssGBLUP, but scenarios using BLUP and GBLUP evaluations were also simulated for comparison. All three methods of selection were implemented using Henderson’s mixed model equation [18].

The assumed linear model is:

$$\mathbf{y}=\mu +\mathbf{Z}\mathbf{a}+\mathbf{e},$$

where $\mathbf{y}$ is the vector of phenotypes, $\mu$ is the overall mean, $\mathbf{a}$ is the vector of polygenic breeding values distributed as $\mathrm{N}({\bf{0}},{\mathbf{{\mathcal{G}}}}{\upsigma}_{a}^{2})$ with $\mathbf{Z}$ being the corresponding incidence matrix, and $\mathbf{e}$ the vector of residual deviations distributed as $\mathrm{N}({\bf{0}},\mathbf{I}{\upsigma }_{e}^{2})$. $\mathbf{{\mathcal{G}}}$ is the genetic relationship matrix associated with the method of evaluation, and $\mathbf{I}$ is an identity matrix. All candidates were assumed to have one phenotypic record available at the time of selection and genotyping, hence they could be in their own genetic evaluation or as a criterion to decide which candidates were to be genotyped.

The BLUP, GBLUP and ssGBLUP evaluation methods are defined by the $\mathbf{{\mathcal{G}}}$ matrix used in the evaluation. For the BLUP evaluation, it is assumed that no genotype information is known for any animals and the relationship matrix is the numerator relationship matrix ($\mathbf{A}$) calculated using pedigree information [18, 19]. For GBLUP, all individuals included in the evaluation are genotyped and the relationship matrix is the genomic relationship matrix ($\mathbf{G}$) calculated using the SNP genotypes [20]. Finally, ssGBLUP assumes that only a proportion of the individuals are genotyped, so the calculation of the relationship matrix ($\mathbf{H}$) combines pedigree and dense SNP information [14]. In this study, the $\mathbf{G}$ matrix was calculated using the second method proposed by VanRaden [20], and the inverse of the $\mathbf{H}$ matrix was calculated by joining the $\mathbf{A}$ matrix (including all individuals, genotyped and ungenotyped) with the $\mathbf{G}$ matrix (including the genotyped individuals only) as described by Legarra et al. [14]. In addition, prior to joining the $\mathbf{A}$ and $\mathbf{G}$ matrices to form $\mathbf{H}$, the $\mathbf{G}$ matrix was adjusted to make it ‘compatible’ with the $\mathbf{A}$ matrix, as ${\mathbf{G}}_{\mathrm{adj}}=a+b*\mathbf{G}$, where $a$ and $b$ were calculated as in Legarra et al. [14].

The selection scheme simulated here assumed multiple discrete generations of selection. Hence, at a given generation, the genetic evaluation of the current candidates to be selected was done with a model including them plus all the information (phenotypes and available genotypes) of animals from previous generations (i.e., the phenotype and genotype information increased with the number of generations).

For the period of selection (G0–G8), the candidates were selected assuming standard truncation based on the genomic breeding values calculated using the linear model described above.

### Scenarios compared

The ssGBLUP scenarios, which were compared here, included eight proportions of candidates being genotyped, three genotyping strategies to select the candidates to be genotyped, based on two ranking criteria. In addition, the BLUP and GBLUP scenarios were also included as they are equivalent to the situations where none or all candidates are genotyped and can be considered as the respective lower and upper limit for the expected performance of the ssGBLUP scenarios.

The proportion ($p$) of candidates being genotyped used in the ssGBLUP scenarios were 10, 20, 30, 40, 50, 60, 70 or 80%. In addition, BLUP and GBLUP are equivalent to scenarios with 0 and 100% genotyped candidates, respectively. The proportions of genotyped candidates were the same for males and females.

The three genotyping strategies were: (i) RANDOM, where the proportion of candidates to be genotyped were chosen at random from all those available; (ii) TOP, where the genotyped animals were the $p$ proportion of candidates (within sex) with the best criterion; and (iii) EXTREME, where the genotyped animals were those with the best $p/2$ and worse $p/2$ criterion. The criterion used to select the candidates to be genotyped with TOP and EXTREME was either: (i) their own phenotypic performance or, (ii) their estimated breeding values (EBV) calculated using BLUP evaluation which included the candidates’ own performance record.

The magnitudes for some other assumptions/parameters needed in the simulation that were related to population structure (e.g. size, male–female ratio, discrete generations) and the genetic architecture (h^2^, LD pattern) were fixed to a given value. This was done in order to focus the study on the main goal about testing the effect of genotyping strategy and the proportion of genotyped candidates on the performance of ssGBLUP in a population with large paternal families and small maternal families (as in sheep populations). We expect that the magnitude of these parameters should have a relative minor impact on the results and that the conclusions drawn here should be general enough so that they may extrapolate to some degree the changes on these parameters.

### Criteria for the comparison

The criteria used for the comparison between scenarios were the cumulative genetic gain ($g$), reliability (${\mathrm{r}}^{2}$) of the genomic estimated breeding values (GEBV) and the retained genetic variance ${\upsigma }_{a}^{2}$.

The cumulative genetic gain was relative to G0 (i.e., TBV were rebased, so the mean at G0 is equal to 0 for all scenarios), corresponding to the first generation where some candidates were ungenotyped and ssGBLUP needed to be implemented. The reliability of the GEBV is the square of the Pearson’s correlation between the GEBV and the TBV in the candidate group at a given time of selection. The retained genetic variance at a given generation is the variance of TBV of all candidates born in that generation.

Because BLUP and GBLUP are equivalent to the scenarios with 0 and 100% of candidates being genotyped, they can be considered to be the respective lower and upper limit of the expected performance of ssGBLUP. Hence, a parameter denoted as Efficiency ($\mathrm{E}$) was defined to compare the different scenarios using ssGBLUP. Basically, $\mathrm{E}$ is the proportion of the extra benefit of GBLUP over BLUP, which is realised on the ssGBLUP scenario (i.e., $\mathrm{E}=\frac{\left(\mathrm{ssGBLUP}-\mathrm{BLUP}\right)}{\left(\mathrm{GBLUP}-\mathrm{BLUP}\right)}$). Therefore, when $\mathrm{E}=1$, it means that the ssGBLUP having a $p$ proportion of genotyped candidates yields the same extra performance as when having all candidates genotyped. On the contrary, when $\mathrm{E}=0$, the ssGBLUP has no advantage over BLUP evaluation where none of the candidates are genotyped. This $\mathrm{E}$ parameter was calculated for the cumulated genetic gain and the reliability at each generation (e.g. $\mathrm{E}$ for gain at generation x for scenario y, would be the extra gain of the scenario y over the BLUP scenario relative to the extra gain achieved with GBLUP).

## Results

This study evaluated the performance of BLUP, GBLUP and 40 ssGBLUP scenarios across three genotyping strategies, eight proportions of genotyped candidates, and two criteria for ranking the candidates to be genotyped. All the results for the different scenarios are in Additional file 2: Tables S1 to S8.

### Benefit of GBLUP when all candidates are genotyped

**Fig. 1:**
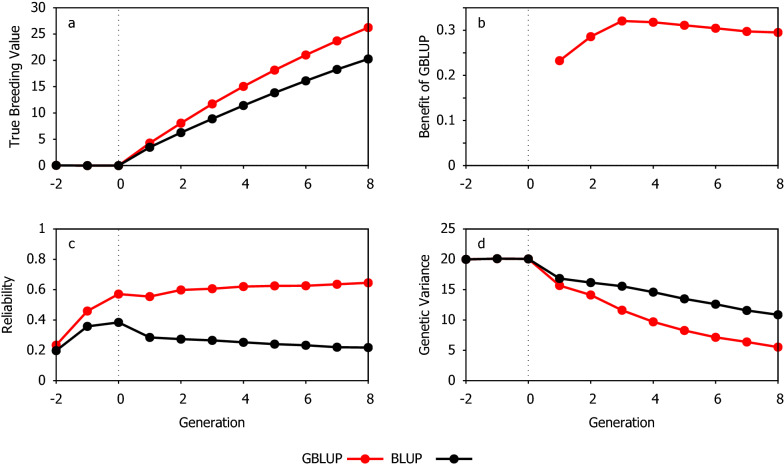
Comparison of the response to selection when using GBLUP (red lines) and BLUP (black lines) evaluation over all generations. **a** Cumulative genetic gain, **b** Extra gain from GBLUP over BLUP, **c** Reliability of GEBV and **d** Remaining genetic variance

The response to selection when assuming that all the animals are genotyped is shown in Fig. 1. Over the generations of selection, GBLUP performed substantially better than BLUP due to a greater reliability of the EBV. The difference in reliability between the two methods increased over generations, such that, at G8, the reliability with GBLUP was close to threefold the value observed with BLUP. However, the benefit of GBLUP remained relatively constant over all generations with selection, with the genetic gain of GBLUP consistently between 23 and 32% higher than that of BLUP. This reflected the fact that the genetic variance decreased faster with GBLUP.

### Benefit of ssGBLUP when only a proportion of candidates are genotyped

**Fig. 2:**
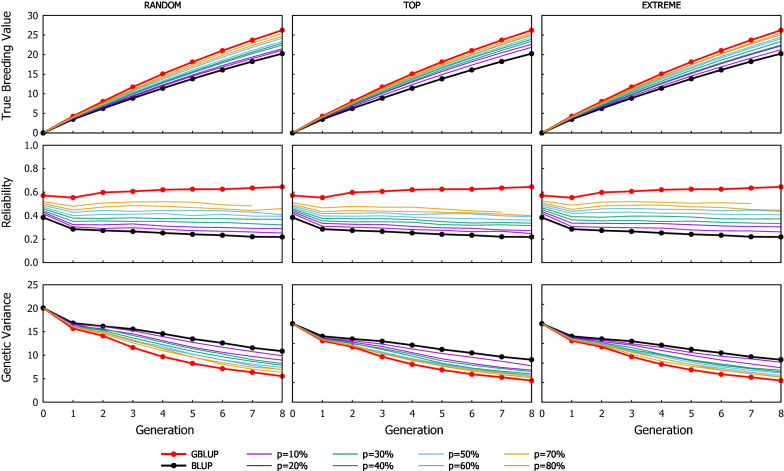
Response to selection over generations for the ssGBLUP scenarios using the three genotyping strategies (RANDOM: left column, TOP: middle column and EXTREME: right column), when the proportion of the genotyped candidates was chosen based on phenotypes. Graphs in the top row show the cumulative genetic gain, graphs in the middle row show the reliability, and graphs in the bottom row show the genetic variance. Results from GBLUP and BLUP are also shown as they are the respective upper and lower limits of ssGBLUP, equivalent to the situations where 100% and 0% of the candidates are genotyped

**Fig. 3:**
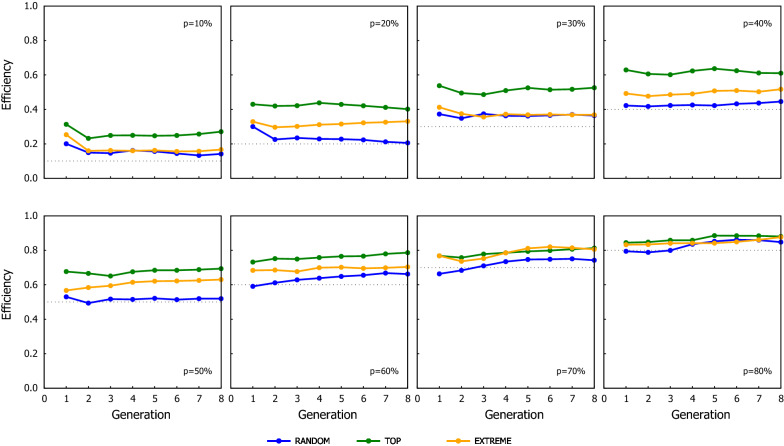
Efficiency of the ssGBLUP scenarios in terms of their cumulative genetic response using three genotyping strategies, when the proportion of the genotyped candidates was chosen based on phenotypes. The dotted line in each graph indicates the proportion of candidates that were genotyped in the corresponding scenario shown in the graph

**Fig. 4:**
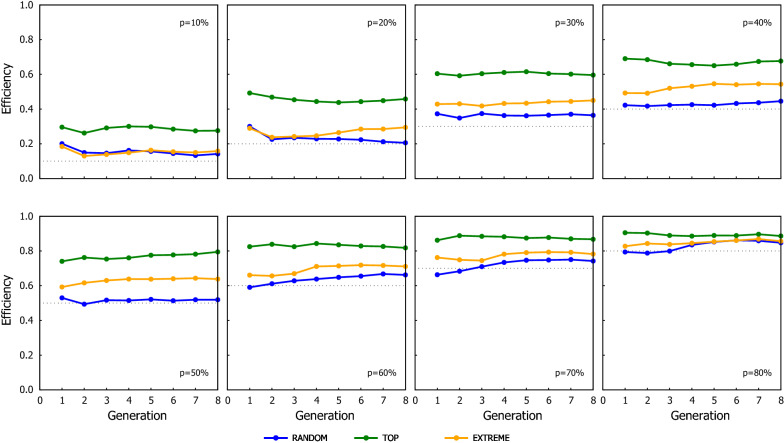
Efficiency of the ssGBLUP scenarios in terms of their cumulative genetic response using three genotyping strategies, when the proportion of the genotyped candidates was chosen based on estimated breeding values. The dotted line in each graph indicates the proportion of candidates that were genotyped in the corresponding scenario shown in the graph

The response to selection for the different scenarios of ssGBLUP when the proportion of the genotyped candidates were preselected based on their phenotype is shown in Fig. 2 (results for the scenarios where the genotyping criterion was their EBV are in Additional file 2: Table S1). As expected, the cumulated genetic gain and the GEBV reliability increased as the proportion of candidates being genotyped increased, which also coincided with a greater loss of genetic variance. Figures 3 and 4 show the Efficiency ($\mathrm{E}$) on realising genetic gain for the ssGBLUP schemes using the candidates’ phenotype or EBV as the genotyping criterion, respectively. The $\mathrm{E}$ tended to be relatively consistent over generations, showing that the value of genotyping a proportion of the candidates to attain the extra genetic gain expected from GBLUP does not improve or degrade with generations. The criterion to rank candidates to be genotyped had an impact on the performance of ssGBLUP, especially with TOP where the use of EBV resulted in 10% (average from values reported in Additional file 2: Tables S5 and S6) more Efficiency in genetic gain compared to using phenotype as the genotyping criterion. The EBV as the genotyping criterion was also beneficial with EXTREME at intermediate $p$ (30–50%).

Within a given proportion of genotyped candidates, the TOP scheme consistently achieved the greatest genetic gain while RANDOM yielded the smallest one (see Figs. 3 and 4). TOP with EBV and $p=20\%$ almost doubled the extra gain obtained with RANDOM (i.e. 96 and 82% greater benefit with TOP based on EBV and on phenotypes, respectively). The behaviour of EXTREME tended to be in between, with its performance being closer to RANDOM with small $p$ but becoming as good as TOP when $p$ increased.

As expected, the performance of ssGBLUP improved as the proportion of genotyped candidates increased, but the increment in performance as $p$ increased occurred at a relatively slow rate. This was particularly true for RANDOM and EXTREME, where the benefit of ssGBLUP was almost linear with the proportion of genotyped candidates: genotyping x% of the candidates achieved around x% of the observed extra gain of GBLUP over BLUP. The Efficiency of the TOP strategy increased slightly faster with an intermediate proportion of genotyped candidates. For $p$ = 30 and 50%, TOP realised 51 and 68% of the maximum extra gain achievable with GBLUP, RANDOM realized 37 and 52%, and EXTREME realized 37 and 61% for the respective proportions (see Fig. 3 and Additional file 2: Table S5). The results for the three genotyping strategies become more similar at high $p$ (~ 80%), which it is not surprising as they are expected to converge to the GBLUP performance when all candidates are genotyped (i.e. $p$ = 100%). These results are different from previous studies, which reported that most of the maximum gain could be achieved by genotyping a relatively small proportion of candidates [21, 22].

**Fig. 5:**
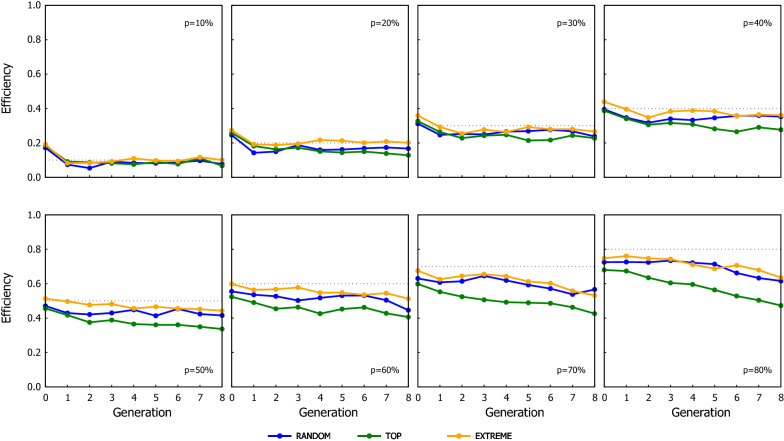
Efficiency of the ssGBLUP in term of their overall reliability (combining both genotyped and ungenotyped animals) using three genotyping strategies, when the proportion of the genotyped candidates was chosen based on phenotypes. The dotted line in each graph indicates the proportion of candidates that were genotyped in the corresponding scenario shown in the graph

**Fig. 6:**
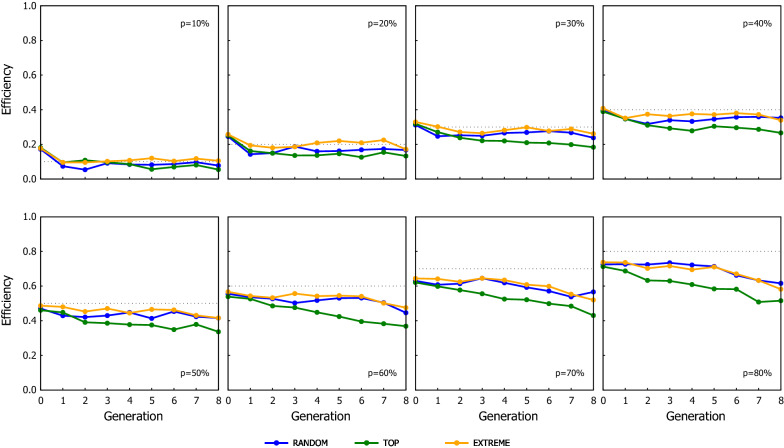
Efficiency of ssGBLUP in term of their overall reliability (combining both genotyped and ungenotyped animals) using three genotyping strategies, when the proportion of the genotyped candidates was chosen based on estimated breeding values. The dotted line in each graph indicates the proportion of candidates that were genotyped in the corresponding scenario shown in the graph

The GEBV reliability for the different ssGBLUP schemes expressed as Efficiency is shown in Figs. 5 and 6. Similarly, as with the genetic gain, the improvement in reliability observed with the ssGBLUP schemes was almost linear with $p$*,* but the ranking of the genotyping strategies changed with EXTREME yielding the highest GEBV reliability, followed by RANDOM, and TOP being the strategy with the lowest GEBV reliability. The fact that RANDOM had consistently a greater reliability than TOP, seems contradictory considering that the simulation assumed discrete generations, so the advantage of a scenario over another should only be due to the GEBV being better estimated.

**Fig. 7:**
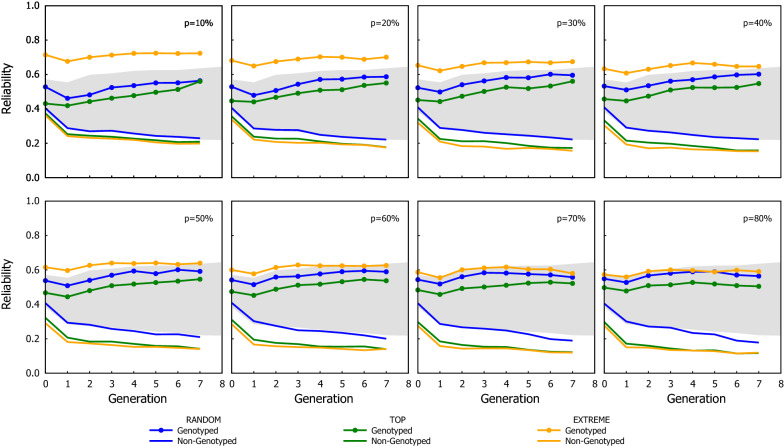
Reliability of ssGBLUP for group of genotyped and ungenotyped candidates using three genotyping strategies, when the proportion of the genotyped candidates was chosen based on phenotypes. The borders of the grey area are the reliability observed with the GBLUP (upper border) and the BLUP (lower border)

**Fig. 8:**
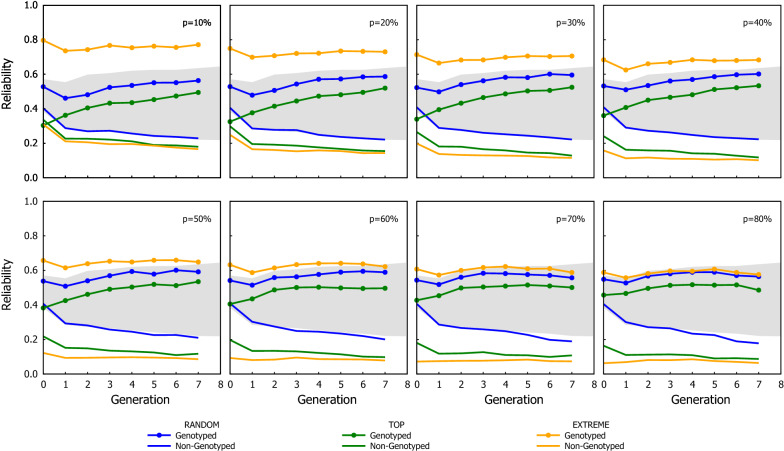
Reliability of ssGBLUP for group of genotyped and ungenotyped candidates using three genotyping strategies, when the proportion of the genotyped candidates was chosen based on estimated breeding values. The borders of the grey area are the reliability observed with the GBLUP (upper border) and the BLUP (lower border)

To better understand the results, the GEBV reliability of genotyped and ungenotyped candidates were re-calculated separately (see Figs. 7 and 8). On the one hand, the reliability of the genotyped candidate group increased over generations (as with GBLUP), and the trends in the reliability between genotyping strategies were the same but their differences were accentuated and became even larger than when the reliability was calculated with both genotyped and ungenotyped candidates together: EXTREME had the highest reliability, followed by RANDOM, and TOP had the lowest reliability (in fact the reliability of genotyped candidates with EXTREME was even greater than that observed with the GBLUP scheme). On the other hand, the reliability of ungenotyped candidates decreased over generations and the best genotyping strategy was RANDOM, followed by TOP and EXTREME. Across all scenarios, the GEBV reliability of genotyped candidates was more than two or three folds the GEBV reliability of the ungenotyped candidates.

**Fig. 9:**
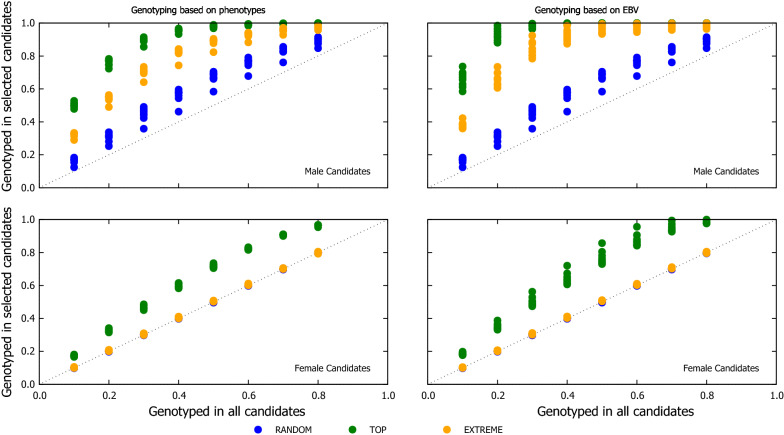
Proportion of the genotyped males (top) and females (bottom) candidates which were selected as parents of the next generation using three genotyping strategies, when the proportion of the genotyped candidates was chosen based on phenotypes (left) or EBV (right). Each point is the value for one replicate (out of 100) across the eight proportions of genotyped candidates (i.e. 10, 20, 30, 40, 50, 60, 70 and 80%). A value of 1 on the y-axis means that all genotyped candidates of a given sex were selected and 0 means no genotyped candidate was selected

A major difference between the genotyping strategy schemes was the proportion of genotyped candidates that were finally selected. TOP tended to have the highest proportion of candidates selected while RANDOM had the lowest proportion of selected candidates, which were genotyped (Fig. 9). Hence, the highest reliability of RANDOM seems to be achieved for candidates that have a low chance to be selected, and therefore, the lower gain is achieved compared with the TOP genotyping scheme.

## Discussion

In this study, we assessed the effect of genotyping strategies and the proportion of genotyped candidates on the cumulated genetic gain when using ssGBLUP evaluation. The results from the simulation study showed that the greatest genetic gain over several generations of selection is achieved with TOP, followed by EXTREME and RANDOM. The extra genetic gain of TOP over standard BLUP was almost double the value attained by RANDOM. The performance of EXTREME was close to RANDOM when the proportion of genotyped candidates was low, but it improved as the proportion increased. The choice of the genotyping criterion had an impact on the genetic gain of the ssGBLUP, with TOP that used EBV yielding a greater benefit than when phenotype was used as the genotyping criterion. However, when comparing their GEBV reliability, the ranking of the strategies changed with EXTREME having the highest reliability, followed by RANDOM, with TOP being the strategy yielding the lowest reliability. The ranking of the genotyping strategies showed the same trend when considering only the group of genotyped candidates. The reliabilities were re-estimated as the square of the Spearman’s rank correlation to test whether the results were due to any potential bias due to a scale effect. The results had the same trend as when using the Pearson’s correlation (i.e. EXTREME > RANDOM > TOP), which suggests that the values on the reliabilities are not an artefact due to differences on the scale (results not shown).

Several published studies have reported the effect of genotyping strategies on GEBV reliability/accuracy using simulation or real data (e.g. [23–26]). Although they tend to consider GBLUP scenarios (where the aim of the genotyping strategy is to select the reference population to be genotyped and phenotyped, but all candidates are assumed to be genotyped), it has frequently been shown that selection of the best candidates to be genotyped leads to the lowest accuracy/reliability on the candidates’ GEBV, with the EXTREME strategy having the highest accuracy/reliability. Such findings are consistent with our results with respect to the ranking of the genotyping strategies in terms of their GEBV reliability. Early studies using the QTL mapping methodology have shown that selective genotyping can improve the power of QTL detection by improving the precision of their estimated effect [27]. Similarly, the impact of selective genotyping on the GEBV reliability can be explained by observing that the error variance of the regression coefficient estimate when fitting a simple linear regression $\mathbf{y}=b\mathbf{x}$ (where $\mathbf{y}$ and $\mathbf{x}$ are centered so their mean is zero) is proportional to the inverse of $\mathbf{x}\mathbf{^{\prime}}\mathbf{x}$ [28]. Then, selecting candidates to be genotyped from both tails of the distribution (as with EXTREME using own phenotype or EBV) would likely increase the proportion of genotyped individuals with opposite genotypes for the SNPs that are associated with the trait. This would increase the magnitude of $\mathbf{x}\mathbf{^{\prime}}\mathbf{x}$, thus leading to more precise SNP effect estimates (thus greater power of QTL detection) and, ultimately, improving the GEBV reliabilities. Conversely, selective genotyping from one tail of the distribution (as with TOP) will select candidates which are more alike (i.e. having the same genotype for the relevant SNPs), thus reducing the magnitude of $\mathbf{x}\mathbf{^{\prime}}\mathbf{x}$ and negatively impacting the GEBV reliabilities.

However, the observation that the greatest genetic gain is not achieved by the strategy with the highest GEBV reliability is counter-intuitive. Our simulations assumed discrete generations, so the differences in cumulated gain should only be due to how well the GEBV were estimated. A recent study assessed the effect of genotyping strategies under ssGBLUP scenarios similar to ours, where only a proportion of the current candidates are genotyped [21]. Under very different assumptions, the authors also found that their scheme, which is equivalent to TOP, yielded the greatest cumulated genetic gain, although the genotyping scheme did not lead to the highest GEBV accuracy. Similarly, Granleese et al. [22] also studied the effect of genotyping with ssGBLUP, but they mainly assessed the effect of different genotyping proportions (assuming a strategy similar to TOP) and the benefit of genotyping male or female candidates. Such disparity between GEBV reliability and genetic gain can be explained by the fact that the enhanced reliability from ssGBLUP is mainly on the genotyped individuals, and the genotyping strategy dictates which candidates benefit from it. Whereas the genotyping strategy has a significant impact on the GEBV reliability, the differences between the genotyped and the ungenotyped candidates are substantially larger than the differences between genotyping strategies. The reliability observed here across genotyping strategies were at most one-fold higher/lower between each other; but the reliabilities of genotyped candidates were two- to threefolds higher than that of ungenotyped candidates (see Figs. 7 and 8).

Hence, when only a proportion of the candidates are genotyped, the optimality to maximise gain is not only about how much the GEBV reliability increases, but also which candidates benefit from the enhanced reliability. For instance, on the one hand, a candidate with very poor performance/EBV probably would be too far below the ranking order to have a significant chance to be selected, so attempting to estimate its GEBV with higher accuracy (by genotyping the candidate itself) would likely not change any preliminary selection decision. On the other hand, a candidate with good performance/EBV would likely rank high, so genotyping the candidate to improve its GEBV reliability may prove beneficial when comparing with other candidates of similar selective advantage. In our study, the TOP scheme genotypes the candidates with the highest phenotype/EBV, meaning that they are also likely to have the highest genetic merit (so increasing the reliability of candidates with a high chance of having high GEBV and being selected). However, the genotyping protocols used with RANDOM and EXTREME mean that some genotyped candidates may have a poor genetic merit (and lower GEBV and less chance of being selected). Hence, the selected candidates chosen with TOP are more likely to be based on more reliable GEBV than with RANDOM and EXTREME with low $p$ (Fig. 9 shows that TOP has a greater proportion of genotyped candidates that are selected, confirming that selection decisions were further aided with more accurate GEBV). This would explain why TOP achieves the greatest genetic gain, although its GEBV reliabilities are, on average, lower than those obtained with RANDOM or EXTREME. The practical implication is that the overall GEBV reliability, when not all candidates are genotyped, is not an absolute and sufficient criterion for determining which scheme would maximise genetic gain over multiple generations.

Our conclusions on the ranking of the selection strategies to maximise gain agree with those reported by Howard et al. [21], but not when considering the effect of the proportion of genotyped candidates. Howard et al. [21] concluded that most of the benefit of the genomic evaluation over standard BLUP is achieved with a low proportion of genotyped animals. For instance, their cattle scenario with dense genotyping has some similar assumptions to our RANDOM and TOP scenarios based on EBV so the results from their Fig. 1 are, somewhat, comparable to our results for the cumulated gain at G8. Our results for TOP based on EBV in Efficiency to achieve extra cumulated genetic gain at G8 were 46, 68, 82 and 89% of the maximum achievable with GBLUP when the proportions of genotyped candidates were 20, 40, 60 and 80%, respectively (see Additional file 2: Table S6). However, their TOP scenario realised substantially greater benefit averaging ~ 73, ~ 85, ~ 91 and ~ 93% for the respective proportions (NB. their values that we report here are approximated as they were calculated based on the visual inspection of their Fig. 1). In fact our results for TOP using EBV was much closer to their RANDOM scenario (i.e. ~ 45, ~ 57, ~ 75 and ~ 85%, respectively). Similarly, the results from Granleese et al. [22] were also as large as those reported by Howard et al. [21], where genotyping only 20% of the TOP candidates would yield as much as 80% of the maximum benefit obtained when all candidates are genotyped.

The disagreements between our results and those from Howard et al. [21] can be partly explained by differences in the assumptions made in our and their simulations. A likely reason may be related to their assumptions on overlapping generations and that any ungenotyped candidate which is selected is genotyped post-selection (our assumption was discrete generations and that selected ungenotyped candidates were not genotyped after selection). In a breeding scheme with overlapping generations, the genetic progress over time arises from two selection processes: (i) the gain from selecting the best replacements; and (ii) the gain from culling the worst current parents. Hence, for a specific ssGBLUP scheme with a proportion $p$ of genotyped candidates, their process of selecting replacements was done with $p$ candidates being genotyped, but the culling process was effectively done with 100% of the parents being genotyped (as in GBLUP) regardless of the ssGBLUP scheme. The consequence of having all selected candidates being genotyped post-selection makes the results for all schemes more alike and closer to GBLUP and thus increasing their relative performance, which would explain why Howard et al. [21] concluded that up to 73% of the maximum benefit from genomic prediction can be obtained by genotyping as little as 20% of the candidates. However, if selected ungenotyped candidates were not to be genotyped at a later stage (in populations with overlapping generations), the enhanced GEBV reliability from the ssGBLUP evaluation would be restricted to only the genotyped parents. Then, the genetic progress from culling parents would depend on $p$, and the benefit of ssGBLUP at low $p$ would be substantially reduced (as observed in our study). There were other notable differences in the assumptions between their and our simulations, which may explain some of the differences, and their impact on the response to selection needs to be properly assessed.

In practice, selected populations most likely have an overlapping population structure, so the post-selection genotyping of any selected ungenotyped animal would be an attractive decision to achieve the extra benefit reported by Howard et al. [21]. However, such a decision may result in the actual proportion of candidates being genotyped (and its cost) ending up to be substantially higher than what the breeder may have originally planned. For instance, our simulation assumed a selection rate of 10% for males and 50% for females, meaning that the final proportion of genotyped candidates would need to equal at least these values. Then, for $p$ = 10%, the final number of genotyped females would be at least (but likely higher) 5 times more than what it was originally planned. Similarly, examining the scenario from Howard et al. [21], which assumes a cattle population with 20% of the animals being genotyped, the final proportion of genotyped candidates during the three generations of GS were 41.5% (1245 out of 3000) and 33.6% (1008 out of 3000) for their equivalent RANDOM and TOP strategies, respectively (see their Table 1). This means that the number of animals to be genotyped could be twice as large as planned, and the associated cost would double too. Hence, from an economical point of view, the post-selection genotyping of any ungenotyped selected candidate may be advisable in schemes with a high intensity of selection, to ensure that the proportion of selected candidates which are still ungenotyped remains relatively low. Generally, this is the case for the selection of male candidates, and genotyping of all selected males may prove an attractive cost benefit practice.

Granleese et al. [22] also reported optimistic conclusions about the benefit of ssGBLUP at low $p$ assuming overlapping generations, but the main reason is not the same as in Howard et al. [21] since the selected ungenotyped candidates were not genotyped post selection. A more plausible explanation for their benefit at low $p$ may be that their simulation approximates the genetic effect/genomic evaluation with the infinitesimal model (whereas our simulation assumed a finite number of loci in LD and a true ssGBLUP evaluation). This assumption ignores changes in the LD pattern and the size/structure of the reference population, thus the GEBV reliability remains fairly more constant over generations and genotyping scenarios. Such discrepancies in assumptions resulted in different trends for the GEBV reliability of genotyped and non-genotyped candidates, thus the difference between these two groups are greater in our study. In fact, the ratio of reliabilities for the group of genotyped to non-genotyped candidates reported by Granleese et al. [22] was 0.155 [${\left(0.71/0.57\right)}^{2}$, see their Table 2] compared to 0.242 observed in our TOP scheme with $p$ = 20% and EBV as the preselection criterion [$\left(0.451/0.186\right)$ using the average of reliabilities values shown in Additional file 2: Table S7], which explains why we observed much less benefit at low $p$*.* Conversely, our assumption of a finite number of loci in the genome has shown faster decline in the genetic variance than that observed in real selection programmes [29], thus further studies may still be needed to better assess the true benefit of ssGBLUP at low $p$.

Hence, if only a proportion of the candidates is to be genotyped, the consensus conclusion (from this study and that from Howard et al. [21]) dictates to prioritise the genotyping of candidates which are more likely to be selected. This raises the need of a priori EBV which can serve as a criterion to rank the candidates, such that the best ones are genotyped (of course, from a simpler evaluation with lower reliability to justify the following ssGBLUP evaluation). Our results showed that using EBV from univariate BLUP to rank candidates to be genotyped can significantly improve the genetic gain of TOP compared to when using the candidates’ own phenotype. The Efficiency of TOP using EBV was up to 17% greater than that of TOP using phenotypes with $p$ = 30%, but it was closer to 10% with other values of $p$, except for $p$ = 80%. The same trend was observed with EXTREME but at lower scale. Hence, the use of a selection index to combine information from more than one trait (EBV or phenotype) (as in [21]) may further improve the beneficial effect of ssGBLUP when selective genotyping is applied, depending on the accuracy of the index to predict the true genetic effect of the trait of interest. However, phenotypes (from the trait itself and/or from correlated ones) may not be available for the candidates themselves (they may be expressed late in life or not measured for any reason). Parental information (e.g., phenotypes, EBV/GEBV) may be used to inform the genotyping process, but as the candidates are not genotyped (and not phenotyped) yet, the ranking of candidates to be genotyped would basically identify the best families to be genotyped (as all candidates from the same family will have the same parental score). Genotyping individuals from a few families may not be the best strategy even if they are the best ones. Performance of genomic prediction declines when the candidates belong to families that differ from those included in a training population [30], and it is expected to worsen within a ssGBLUP framework. In addition, restrictions on selected candidates per family to control inbreeding also undermine the Efficiency of the ssGBLUP scheme. Hence, in practice, if phenotype information is not available when the decision of genotyping is to be taken, a mixture of the TOP and RANDOM strategies is likely to be the only possible option, where the best families are selected but few individuals within the family are selected at random. This could have a serious impact on the Efficiency of the scheme and, the proportion of genotyped candidates may need to be much higher in order to ensure a significant impact of the genomic prediction in the selection scheme. Clearly the availability of candidates’ own performance records (phenotypic records on the trait of interest or other trait(s) genetically correlated to the former one) are needed to ensure that the genotyping criterion can distinguish candidates from the same family so that efficient strategies can be implemented when only a proportion of the candidates are to be genotyped.

## Conclusions

When only a proportion of the candidates are to be genotyped, TOP is the best strategy to ensure that genetic gain is maximised, since it prioritises the genotyping of candidates which are more likely to be selected. However, in this study, the Efficiency of ssGBLUP on realising the extra genetic gain of GBLUP does not improve as fast with proportion of genotyped candidates as reported in other studies. The choice of EBV rather than phenotypes to rank candidates to be genotyped improves the extra genetic gain of ssGBLUP using TOP. Since in our study, where not all candidates were genotyped, the highest genetic gain was not achieved by the strategy with the greatest GEBV reliability, the latter parameter cannot be considered as an absolute and sufficient criterion to determine which scheme would maximise genetic gain over multiple generations. Hence, to assess the best strategy to maximise gain in a specific situation, any feasibility study should not be restricted to quantifying the gain in GEBV reliability for the alternative schemes, but also their expected genetic progress.

## Supplementary Information

**Additional file 1: Figure S1.** Histogram of the minor allele frequency (MAF) at G(− 2) for all loci chosen to be QTL or as part of the SNP panel across all 100 replicates.

**Additional file 2: Table S1.** Cumulated genetic gain, genetic variance and reliability observed in the GBLUP and BLUP scenarios over generations. **Table S2.** Cumulated genetic gain over generations for all ssGBLUP scenarios. **Table S3.** Genetic variance over generations for all ssGBLUP scenarios. **Table S4.** Reliability over generations for all ssGBLUP scenarios. **Table S5.** Efficiency of ssGBLUP in terms of their cumulative genetic response and reliability for all ssGBLUP scenarios when the genotyping criterion was the candidates’ own phenotypes. **Table S6.** Efficiency of ssGBLUP in terms of their cumulative genetic response and reliability for all ssGBLUP scenarios when the genotyping criterion was the candidates’ estimated breeding values. **Table S7.** GEBV reliability estimates observed for all ssGBLUP scenarios. **Table S8.** Proportion of genotyped candidates that were selected as parents for all ssGBLUP scenarios.

## Data Availability

Not applicable.

## References

[1] Meuwissen THE, Hayes BJ, Goddard ME (2001). Prediction of total genetic value using genome-wide dense marker maps. Genetics.

[2] Tsai HY, Hamilton A, Tinch AE, Guy DR, Bron JE, Taggart JB (2016). Genomic prediction of host resistance to sea lice in farmed Atlantic salmon populations. Genet Sel Evol.

[3] Christensen OF, Madsen P, Nielsen B, Ostersen T, Su G (2012). Single-step methods for genomic evaluation in pigs. Animal.

[4] Hayes BJ, Bowman PJ, Chamberlain AJ, Goddard ME (2009). Invited review: Genomic selection in dairy cattle: progress and challenges. J Dairy Sci.

[5] VanRaden PM, Van Tassell CP, Wiggans GR, Sonstegard TS, Schnabel RD, Taylor JF (2009). Invited review: Reliability of genomic predictions for North American Holstein bulls. J Dairy Sci.

[6] Wolc A, Kranis A, Arango J, Settar P, Fulton JE, O’Sullivan NP (2016). Implementation of genomic selection in the poultry industry. Anim Front.

[7] Knol EF, Nielsen B, Knap PW (2016). Genomic selection in commercial pig breeding. Anim Front.

[8] Riggio V, Abdel-Aziz M, Matika O, Moreno CR, Carta A, Bishop SC (2014). Accuracy of genomic prediction within and across populations for nematode resistance and body weight traits in sheep. Animal.

[9] García-Ruiz A, Cole JB, VanRaden PM, Wiggans GR, Ruiz-López FJ, Van Tassell CP (2016). Changes in genetic selection differentials and generation intervals in US Holstein dairy cattle as a result of genomic selection. Proc Natl Acad Sci USA.

[10] Doublet A-C, Croiseau P, Fritz S, Michenet A, Hozé C, Danchin-Burge C (2019). The impact of genomic selection on genetic diversity and genetic gain in three French dairy cattle breeds. Genet Sel Evol.

[11] Gianola D, de los Campos G, Hill WG, Manfredi E, Fernando R (2009). Additive genetic variability and the Bayesian alphabet. Genetics.

[12] Gianola D (2013). Priors in whole-genome regression: the Bayesian alphabet returns. Genetics.

[13] Hayes BJ, Visscher PM, Goddard ME (2009). Increased accuracy of artificial selection by using the realized relationship matrix. Genet Res.

[14] Legarra A, Christensen OF, Aguilar I, Misztal I (2014). Single Step, a general approach for genomic selection. Livest Sci.

[15] Christensen OF, Lund MS (2010). Genomic prediction when some animals are not genotyped. Genet Sel Evol.

[16] Legarra A, Aguilar I, Misztal I (2009). A relationship matrix including full pedigree and genomic information. J Dairy Sci.

[17] Misztal I, Aggrey SE, Muir WM (2013). Experiences with a single-step genome evaluation. Poult Sci.

[18] Henderson CR (1984). Applications of linear models in animal breeding.

[19] Henderson CR (1976). A simple method for computing the inverse of a Numerator Relationship Matrix used in prediction of breeding values. Biometrics.

[20] VanRaden PM (2008). Efficient methods to compute genomic predictions. J Dairy Sci.

[21] Howard JT, Rathje TA, Bruns CE, Wilson-Wells DF, Kachman SD, Spangler ML (2018). The impact of selective genotyping on the response to selection using single-step genomic best linear unbiased prediction. J Anim Sci.

[22] Granleese T, Clark SA, van der Werf JHJ (2019). Genotyping strategies of selection candidates in livestock breeding programmes. J Anim Breed Genet.

[23] Boligon AA, Long N, Albuquerque LG, Weigel KA, Gianola D, Rosa GJM (2012). Comparison of selective genotyping strategies for prediction of breeding values in a population undergoing selection. J Anim Sci.

[24] Pszczola M, Calus MPL (2016). Updating the reference population to achieve constant genomic prediction reliability across generations. Animal.

[25] Jiménez-Montero JA, González-Recio O, Alenda R (2012). Genotyping strategies for genomic selection in small dairy cattle populations. Animal.

[26] Jenko J, Wiggans GR, Cooper TA, Eaglen SAE, Luff WGD, Bichard M (2017). Cow genotyping strategies for genomic selection in a small dairy cattle population. J Dairy Sci.

[27] Darvasi A, Soller M (1992). Selective genotyping for determination of linkage between a marker locus and a quantitative trait locus. Theor Appl Genet.

[28] Snedecor GW, Cochran WG (1967). Statistical methods.

[29] Mulder HA, Lee SH, Clark S, Hayes BJ, van der Werf JHJ (2019). The impact of genomic and traditional selection on the contribution of mutational variance to long-term selection response and genetic variance. Genetics.

[30] Legarra A, Robert-Granié C, Manfredi E, Elsen JM (2008). Performance of genomic selection in mice. Genetics.

