# Supplementary figures and images for: Effect of genotyping strategies on the sustained benefit of single-step genomic BLUP over multiple generations
Source: Genet Sel Evol. 2022 Mar 18;54:23. doi: 10.1186/s12711-022-00712-y (PMC8931970; doi:10.1186/s12711-022-00712-y)

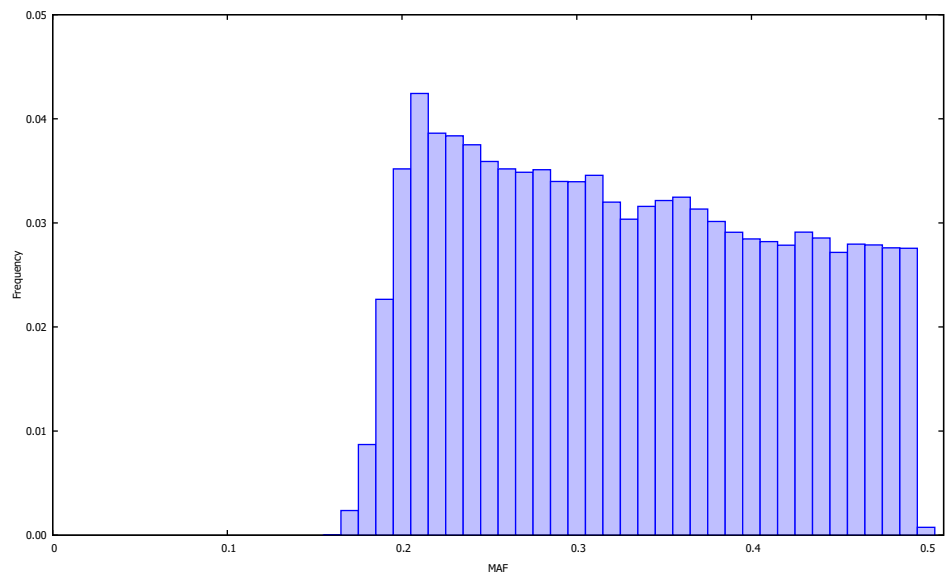

Supplement: Supplementary file 1 — Additional file 1: Figure S1. Histogram of the minor allele frequency (MAF) at G(− 2) for all loci chosen to be QTL or as part of the SNP panel across all 100 replicates. [file 12711_2022_712_MOESM1_ESM.pdf]
