# Supplementary material for: Effect of genotyping strategies on the sustained benefit of single-step genomic BLUP over multiple generations
Source: Genet Sel Evol. 2022 Mar 18;54:23. doi: 10.1186/s12711-022-00712-y (PMC8931970; doi:10.1186/s12711-022-00712-y)
Supplement: Supplementary file 2 — Additional file 2: Table S1. Cumulated genetic gain, genetic variance and reliability observed in the GBLUP and BLUP scenarios over generations. Table S2. Cumulated genetic gain over generations for all ssGBLUP scenarios. Table S3. Genetic variance over generations for all ssGBLUP scenarios. Table S4. Reliability over generations for all ssGBLUP scenarios. Table S5. Efficiency of ssGBLUP in terms of their cumulative genetic response and reliability for all ssGBLUP scenarios when the genotyping criterion was the candidates’ own phenotypes. Table S6. Efficiency of ssGBLUP in terms of their cumulative genetic response and reliability for all ssGBLUP scenarios when the genotyping criterion was the candidates’ estimated breeding values. Table S7. GEBV reliability estimates observed for all ssGBLUP scenarios. Table S8. Proportion of genotyped candidates that were selected as parents for all ssGBLUP scenarios. [file 12711_2022_712_MOESM2_ESM.docx]

**Appendix file 2. Results from the simulation study**

The following tables show the results for the BLUP, GBLUP and ssGBLUP scenarios considered in this study. The ssGBLUP includes 40 scenarios across eight proportions of genotyped candidates (*p*=0.1, 0.2, 0.3, 0.4, 0.5. 0.6 0.7, 0.8), three genotyping strategies (RANDOM, TOP, EXTREME) using either phenotype or EBV for ranking candidates to genotype.

The population structure is composed of 900 individuals (300 males and 600 females). At each generation, 30 males and 300 females were selected to be the parents of the next generation parents. Each male was mated with 10 females and each female produced three offspring (1 male and 2 females).

The trait has a phenotypic variance of 100 and its heritability was 0.2. The genome was composed of 26 chromosomes of one Morgan length. Each chromosome had 1,100 loci: 100 were QTL and 1,000 were part of the SNP panel used to calculate the GRM (i.e. 2,600 QTL and 26,000 SNPs across the genome). The QTL were assumed totally additive and their effects sampled from a normal distribution with mean zero and variance 1, which later was rescaled so the variance for the true breeding values at G(-2) was 20. All individuals have one phenotypic record known at the time of selection and when the decision of which candidate to genotype was taken.

Discrete generations were assumed. The first two generation (G(-2), G(-1)) were carried out under random selection and all individuals were genotyped to create the initial reference population to be used in the GBLUP and ssGBLUP schemes. G0 was the start of the selection scheme and when the selective genotyping (for ssGBLUP) started. Selection was applied across 8 generations (G0-G8).

The results presented are the mean of 100 replicates. The cumulated genetic gain is expressed as deviation from G0, which is the last generation created without selection.

**Table S1: Cumulated genetic gain, genetic variance and reliability observed in the GBLUP and BLUP scenarios across the generations.**

| Gen* | Cumulated Genetic Gain | | Genetic Variance | | Reliability | |
| --- | --- | --- | --- | --- | --- | --- |
|  | GBLUP | BLUP | GBLUP | BLUP | GBLUP | BLUP |
| G(-2) | 0.04 | 0.04 | 20.00 | 20.00 | 0.23 | 0.20 |
| G(-1) | 0.00 | 0.00 | 20.10 | 20.10 | 0.46 | 0.36 |
| G0 | 0.00 | 0.00 | 20.07 | 20.07 | 0.57 | 0.38 |
| G1 | 4.29 | 3.48 | 15.68 | 16.83 | 0.55 | 0.28 |
| G2 | 8.06 | 6.27 | 14.13 | 16.16 | 0.60 | 0.27 |
| G3 | 11.73 | 8.88 | 11.62 | 15.57 | 0.61 | 0.27 |
| G4 | 15.06 | 11.43 | 9.69 | 14.59 | 0.62 | 0.25 |
| G5 | 18.15 | 13.84 | 8.27 | 13.48 | 0.63 | 0.24 |
| G6 | 21.03 | 16.12 | 7.15 | 12.61 | 0.63 | 0.23 |
| G7 | 23.70 | 18.27 | 6.37 | 11.58 | 0.64 | 0.22 |
| G8 | 26.24 | 20.26 | 5.53 | 10.86 | 0.64 | 0.22 |

***:** parents from generations G(-2) and (G-1) were selected at random. The directional selection started at G0

**Table S2. Cumulated Genetic Gain across generations for all ssGBLUP scenarios**

| *p* | gen |  | Genotyping based on phenotype | | Genotyping based on EBV | |
| --- | --- | --- | --- | --- | --- | --- |
|  |  | RANDOM | TOP | EXTREME | TOP | EXTREME |
| 0.1 | G0 | 0.00 | 0.00 | 0.00 | 0.00 | 0.00 |
| 0.1 | G1 | 3.64 | 3.73 | 3.68 | 3.72 | 3.63 |
| 0.1 | G2 | 6.54 | 6.68 | 6.55 | 6.74 | 6.50 |
| 0.1 | G3 | 9.30 | 9.59 | 9.34 | 9.71 | 9.27 |
| 0.1 | G4 | 12.01 | 12.33 | 12.01 | 12.52 | 11.97 |
| 0.1 | G5 | 14.51 | 14.90 | 14.54 | 15.12 | 14.54 |
| 0.1 | G6 | 16.83 | 17.34 | 16.89 | 17.52 | 16.88 |
| 0.1 | G7 | 18.99 | 19.67 | 19.13 | 19.76 | 19.08 |
| 0.1 | G8 | 21.11 | 21.88 | 21.26 | 21.91 | 21.21 |
|  |  |  |  |  |  |  |
| 0.2 | G0 | 0.00 | 0.00 | 0.00 | 0.00 | 0.00 |
| 0.2 | G1 | 3.72 | 3.83 | 3.75 | 3.88 | 3.71 |
| 0.2 | G2 | 6.67 | 7.02 | 6.80 | 7.11 | 6.69 |
| 0.2 | G3 | 9.55 | 10.08 | 9.74 | 10.17 | 9.57 |
| 0.2 | G4 | 12.26 | 13.02 | 12.56 | 13.04 | 12.32 |
| 0.2 | G5 | 14.82 | 15.69 | 15.20 | 15.73 | 14.98 |
| 0.2 | G6 | 17.21 | 18.19 | 17.70 | 18.30 | 17.52 |
| 0.2 | G7 | 19.42 | 20.51 | 20.04 | 20.71 | 19.82 |
| 0.2 | G8 | 21.49 | 22.66 | 22.24 | 23.00 | 22.02 |
|  |  |  |  |  |  |  |
| 0.3 | G0 | 0.00 | 0.00 | 0.00 | 0.00 | 0.00 |
| 0.3 | G1 | 3.78 | 3.92 | 3.81 | 3.97 | 3.83 |
| 0.3 | G2 | 6.89 | 7.16 | 6.94 | 7.33 | 7.04 |
| 0.3 | G3 | 9.95 | 10.27 | 9.89 | 10.60 | 10.07 |
| 0.3 | G4 | 12.75 | 13.28 | 12.78 | 13.65 | 13.00 |
| 0.3 | G5 | 15.40 | 16.10 | 15.43 | 16.49 | 15.71 |
| 0.3 | G6 | 17.91 | 18.65 | 17.94 | 19.09 | 18.29 |
| 0.3 | G7 | 20.28 | 21.08 | 20.27 | 21.54 | 20.68 |
| 0.3 | G8 | 22.44 | 23.41 | 22.47 | 23.82 | 22.95 |
|  |  |  |  |  |  |  |
| 0.4 | G0 | 0.00 | 0.00 | 0.00 | 0.00 | 0.00 |
| 0.4 | G1 | 3.82 | 3.99 | 3.88 | 4.04 | 3.88 |
| 0.4 | G2 | 7.02 | 7.35 | 7.12 | 7.50 | 7.15 |
| 0.4 | G3 | 10.09 | 10.59 | 10.26 | 10.76 | 10.36 |
| 0.4 | G4 | 12.97 | 13.69 | 13.21 | 13.81 | 13.36 |
| 0.4 | G5 | 15.66 | 16.58 | 16.03 | 16.64 | 16.19 |
| 0.4 | G6 | 18.24 | 19.19 | 18.62 | 19.36 | 18.77 |
| 0.4 | G7 | 20.64 | 21.59 | 21.00 | 21.93 | 21.23 |
| 0.4 | G8 | 22.93 | 23.91 | 23.35 | 24.31 | 23.51 |
|  |  |  |  |  |  |  |
| 0.5 | G0 | 0.00 | 0.00 | 0.00 | 0.00 | 0.00 |
| 0.5 | G1 | 3.91 | 4.03 | 3.94 | 4.08 | 3.96 |
| 0.5 | G2 | 7.15 | 7.46 | 7.31 | 7.63 | 7.37 |
| 0.5 | G3 | 10.35 | 10.73 | 10.57 | 11.03 | 10.68 |
| 0.5 | G4 | 13.30 | 13.88 | 13.66 | 14.19 | 13.75 |
| 0.5 | G5 | 16.09 | 16.79 | 16.51 | 17.18 | 16.59 |
| 0.5 | G6 | 18.64 | 19.48 | 19.18 | 19.94 | 19.26 |
| 0.5 | G7 | 21.09 | 22.01 | 21.67 | 22.52 | 21.77 |
| 0.5 | G8 | 23.37 | 24.41 | 24.03 | 25.02 | 24.08 |
|  |  |  |  |  |  |  |
| 0.6 | G0 | 0.00 | 0.00 | 0.00 | 0.00 | 0.00 |
| 0.6 | G1 | 3.96 | 4.07 | 4.03 | 4.15 | 4.02 |
| 0.6 | G2 | 7.36 | 7.62 | 7.50 | 7.77 | 7.44 |
| 0.6 | G3 | 10.67 | 11.01 | 10.81 | 11.23 | 10.79 |
| 0.6 | G4 | 13.75 | 14.18 | 13.97 | 14.49 | 14.01 |
| 0.6 | G5 | 16.63 | 17.13 | 16.86 | 17.44 | 16.92 |
| 0.6 | G6 | 19.34 | 19.88 | 19.53 | 20.19 | 19.65 |
| 0.6 | G7 | 21.90 | 22.51 | 22.07 | 22.76 | 22.16 |
| 0.6 | G8 | 24.22 | 24.96 | 24.47 | 25.16 | 24.52 |
|  |  |  |  |  |  |  |
| 0.7 | G0 | 0.00 | 0.00 | 0.00 | 0.00 | 0.00 |
| 0.7 | G1 | 4.02 | 4.10 | 4.10 | 4.18 | 4.10 |
| 0.7 | G2 | 7.49 | 7.63 | 7.59 | 7.86 | 7.61 |
| 0.7 | G3 | 10.90 | 11.10 | 11.02 | 11.40 | 11.00 |
| 0.7 | G4 | 14.10 | 14.28 | 14.28 | 14.63 | 14.27 |
| 0.7 | G5 | 17.06 | 17.26 | 17.34 | 17.61 | 17.25 |
| 0.7 | G6 | 19.80 | 20.04 | 20.15 | 20.43 | 20.02 |
| 0.7 | G7 | 22.35 | 22.65 | 22.69 | 23.00 | 22.58 |
| 0.7 | G8 | 24.70 | 25.12 | 25.08 | 25.45 | 24.94 |
|  |  |  |  |  |  |  |
| 0.8 | G0 | 0.00 | 0.00 | 0.00 | 0.00 | 0.00 |
| 0.8 | G1 | 4.12 | 4.16 | 4.15 | 4.21 | 4.15 |
| 0.8 | G2 | 7.68 | 7.79 | 7.76 | 7.89 | 7.78 |
| 0.8 | G3 | 11.16 | 11.32 | 11.27 | 11.41 | 11.27 |
| 0.8 | G4 | 14.46 | 14.55 | 14.49 | 14.65 | 14.50 |
| 0.8 | G5 | 17.51 | 17.65 | 17.46 | 17.67 | 17.52 |
| 0.8 | G6 | 20.35 | 20.47 | 20.29 | 20.49 | 20.35 |
| 0.8 | G7 | 22.94 | 23.07 | 22.95 | 23.14 | 22.99 |
| 0.8 | G8 | 25.33 | 25.53 | 25.50 | 25.56 | 25.39 |

**Table S3. Genetic variance across generations for all ssGBLUP scenarios**

| *p* | gen |  | Genotyping based on phenotype | | Genotyping based on EBV | |
| --- | --- | --- | --- | --- | --- | --- |
|  |  | RANDOM | TOP | EXTREME | TOP | EXTREME |
| 0.1 | G0 | 20.07 | 20.07 | 20.07 | 20.07 | 20.07 |
| 0.1 | G1 | 16.61 | 16.40 | 16.53 | 16.67 | 16.80 |
| 0.1 | G2 | 16.10 | 15.74 | 15.87 | 15.84 | 16.04 |
| 0.1 | G3 | 15.22 | 14.85 | 14.93 | 14.86 | 15.46 |
| 0.1 | G4 | 13.97 | 13.60 | 13.98 | 13.64 | 14.37 |
| 0.1 | G5 | 12.74 | 12.45 | 12.67 | 12.16 | 13.16 |
| 0.1 | G6 | 11.69 | 11.48 | 11.75 | 10.98 | 12.07 |
| 0.1 | G7 | 10.76 | 10.44 | 11.06 | 10.02 | 11.12 |
| 0.1 | G8 | 9.87 | 9.33 | 10.20 | 9.18 | 10.14 |
|  |  |  |  |  |  |  |
| 0.2 | G0 | 20.07 | 20.07 | 20.07 | 20.07 | 20.07 |
| 0.2 | G1 | 16.54 | 16.42 | 16.45 | 16.47 | 16.55 |
| 0.2 | G2 | 15.45 | 15.33 | 15.47 | 15.16 | 15.73 |
| 0.2 | G3 | 14.55 | 14.21 | 14.62 | 13.64 | 14.63 |
| 0.2 | G4 | 13.11 | 12.66 | 13.22 | 12.34 | 13.50 |
| 0.2 | G5 | 11.70 | 11.11 | 11.98 | 11.18 | 12.22 |
| 0.2 | G6 | 10.61 | 9.88 | 10.77 | 10.17 | 11.00 |
| 0.2 | G7 | 9.74 | 8.89 | 9.75 | 9.35 | 10.00 |
| 0.2 | G8 | 8.95 | 8.21 | 8.83 | 8.54 | 8.98 |
|  |  |  |  |  |  |  |
| 0.3 | G0 | 20.07 | 20.07 | 20.07 | 20.07 | 20.07 |
| 0.3 | G1 | 16.21 | 16.23 | 16.22 | 16.32 | 16.45 |
| 0.3 | G2 | 15.65 | 14.96 | 15.16 | 15.23 | 15.54 |
| 0.3 | G3 | 14.21 | 13.71 | 13.97 | 13.42 | 14.16 |
| 0.3 | G4 | 12.78 | 12.26 | 12.23 | 11.79 | 12.71 |
| 0.3 | G5 | 11.39 | 10.55 | 10.81 | 10.35 | 11.44 |
| 0.3 | G6 | 10.28 | 9.46 | 9.75 | 9.06 | 10.24 |
| 0.3 | G7 | 9.03 | 8.60 | 8.74 | 8.16 | 9.25 |
| 0.3 | G8 | 8.24 | 7.81 | 8.16 | 7.54 | 8.13 |
|  |  |  |  |  |  |  |
| 0.4 | G0 | 20.07 | 20.07 | 20.07 | 20.07 | 20.07 |
| 0.4 | G1 | 16.23 | 16.18 | 16.22 | 16.21 | 16.24 |
| 0.4 | G2 | 15.26 | 15.03 | 15.02 | 14.83 | 15.39 |
| 0.4 | G3 | 13.68 | 13.48 | 13.42 | 12.95 | 13.81 |
| 0.4 | G4 | 12.25 | 11.77 | 12.09 | 11.32 | 12.44 |
| 0.4 | G5 | 10.85 | 9.97 | 10.59 | 10.09 | 10.93 |
| 0.4 | G6 | 9.67 | 8.73 | 9.43 | 8.98 | 9.70 |
| 0.4 | G7 | 8.70 | 7.90 | 8.55 | 7.92 | 8.61 |
| 0.4 | G8 | 7.85 | 7.26 | 7.76 | 7.20 | 7.67 |
|  |  |  |  |  |  |  |
| 0.5 | G0 | 20.07 | 20.07 | 20.07 | 20.07 | 20.07 |
| 0.5 | G1 | 16.03 | 16.03 | 16.13 | 16.16 | 16.24 |
| 0.5 | G2 | 14.97 | 14.38 | 15.10 | 14.62 | 15.11 |
| 0.5 | G3 | 13.11 | 12.80 | 13.14 | 12.86 | 13.36 |
| 0.5 | G4 | 11.84 | 11.11 | 11.74 | 11.25 | 11.64 |
| 0.5 | G5 | 10.22 | 9.78 | 10.14 | 9.64 | 10.17 |
| 0.5 | G6 | 9.03 | 8.52 | 9.05 | 8.52 | 9.11 |
| 0.5 | G7 | 8.10 | 7.64 | 8.15 | 7.83 | 8.00 |
| 0.5 | G8 | 7.37 | 6.88 | 7.42 | 6.90 | 7.21 |
|  |  |  |  |  |  |  |
| 0.6 | G0 | 20.07 | 20.07 | 20.07 | 20.07 | 20.07 |
| 0.6 | G1 | 16.23 | 16.02 | 16.09 | 15.97 | 15.89 |
| 0.6 | G2 | 14.86 | 14.56 | 14.76 | 14.61 | 14.81 |
| 0.6 | G3 | 13.01 | 12.67 | 13.03 | 12.80 | 13.28 |
| 0.6 | G4 | 11.47 | 10.89 | 11.15 | 10.79 | 11.51 |
| 0.6 | G5 | 10.19 | 9.60 | 9.60 | 9.17 | 10.07 |
| 0.6 | G6 | 8.98 | 8.54 | 8.65 | 8.14 | 8.61 |
| 0.6 | G7 | 7.83 | 7.55 | 7.64 | 7.23 | 7.67 |
| 0.6 | G8 | 6.90 | 6.85 | 6.84 | 6.42 | 6.82 |
|  |  |  |  |  |  |  |
| 0.7 | G0 | 20.07 | 20.07 | 20.07 | 20.07 | 20.07 |
| 0.7 | G1 | 16.06 | 15.97 | 15.97 | 16.02 | 15.97 |
| 0.7 | G2 | 14.90 | 14.52 | 14.67 | 14.28 | 14.38 |
| 0.7 | G3 | 13.28 | 12.36 | 12.77 | 12.23 | 12.56 |
| 0.7 | G4 | 11.32 | 10.65 | 11.14 | 10.61 | 10.99 |
| 0.7 | G5 | 9.59 | 9.31 | 9.62 | 9.07 | 9.62 |
| 0.7 | G6 | 8.42 | 8.25 | 8.27 | 7.89 | 8.22 |
| 0.7 | G7 | 7.47 | 7.39 | 7.30 | 7.12 | 7.21 |
| 0.7 | G8 | 6.96 | 6.53 | 6.48 | 6.22 | 6.35 |
|  |  |  |  |  |  |  |
| 0.8 | G0 | 20.07 | 20.07 | 20.07 | 20.07 | 20.07 |
| 0.8 | G1 | 16.02 | 15.91 | 15.91 | 15.90 | 15.93 |
| 0.8 | G2 | 14.61 | 14.37 | 14.47 | 14.12 | 14.34 |
| 0.8 | G3 | 12.63 | 12.33 | 12.26 | 12.02 | 12.40 |
| 0.8 | G4 | 10.93 | 10.87 | 10.47 | 10.31 | 10.65 |
| 0.8 | G5 | 9.65 | 9.17 | 9.18 | 8.93 | 9.17 |
| 0.8 | G6 | 8.07 | 8.00 | 8.18 | 7.81 | 8.19 |
| 0.8 | G7 | 7.04 | 7.02 | 7.25 | 6.73 | 7.11 |
| 0.8 | G8 | 6.37 | 6.23 | 6.38 | 6.10 | 6.20 |

**Table S4. Reliability across generations for all ssGBLUP scenarios**

| *p* | gen |  | Genotyping based on phenotype | | Genotyping based on EBV | |
| --- | --- | --- | --- | --- | --- | --- |
|  |  | RANDOM | TOP | EXTREME | TOP | EXTREME |
| 0.1 | G0 | 0.42 | 0.42 | 0.42 | 0.42 | 0.42 |
| 0.1 | G1 | 0.30 | 0.31 | 0.31 | 0.31 | 0.31 |
| 0.1 | G2 | 0.29 | 0.30 | 0.30 | 0.31 | 0.30 |
| 0.1 | G3 | 0.30 | 0.29 | 0.30 | 0.30 | 0.30 |
| 0.1 | G4 | 0.28 | 0.28 | 0.29 | 0.28 | 0.29 |
| 0.1 | G5 | 0.27 | 0.27 | 0.28 | 0.26 | 0.29 |
| 0.1 | G6 | 0.27 | 0.26 | 0.27 | 0.26 | 0.27 |
| 0.1 | G7 | 0.26 | 0.27 | 0.27 | 0.25 | 0.27 |
| 0.1 | G8 | 0.25 | 0.25 | 0.26 | 0.24 | 0.26 |
|  |  |  |  |  |  |  |
| 0.2 | G0 | 0.43 | 0.43 | 0.44 | 0.43 | 0.43 |
| 0.2 | G1 | 0.32 | 0.33 | 0.34 | 0.33 | 0.34 |
| 0.2 | G2 | 0.32 | 0.33 | 0.33 | 0.32 | 0.33 |
| 0.2 | G3 | 0.33 | 0.32 | 0.33 | 0.31 | 0.33 |
| 0.2 | G4 | 0.31 | 0.31 | 0.33 | 0.30 | 0.33 |
| 0.2 | G5 | 0.30 | 0.30 | 0.32 | 0.30 | 0.33 |
| 0.2 | G6 | 0.30 | 0.29 | 0.31 | 0.28 | 0.32 |
| 0.2 | G7 | 0.29 | 0.28 | 0.31 | 0.28 | 0.31 |
| 0.2 | G8 | 0.29 | 0.27 | 0.30 | 0.28 | 0.29 |
|  |  |  |  |  |  |  |
| 0.3 | G0 | 0.44 | 0.45 | 0.45 | 0.44 | 0.45 |
| 0.3 | G1 | 0.35 | 0.36 | 0.36 | 0.36 | 0.37 |
| 0.3 | G2 | 0.36 | 0.35 | 0.36 | 0.35 | 0.36 |
| 0.3 | G3 | 0.35 | 0.35 | 0.36 | 0.34 | 0.36 |
| 0.3 | G4 | 0.35 | 0.34 | 0.35 | 0.33 | 0.36 |
| 0.3 | G5 | 0.34 | 0.32 | 0.35 | 0.32 | 0.36 |
| 0.3 | G6 | 0.34 | 0.32 | 0.34 | 0.31 | 0.34 |
| 0.3 | G7 | 0.33 | 0.32 | 0.34 | 0.30 | 0.34 |
| 0.3 | G8 | 0.32 | 0.32 | 0.33 | 0.30 | 0.33 |
|  |  |  |  |  |  |  |
| 0.4 | G0 | 0.46 | 0.46 | 0.47 | 0.46 | 0.46 |
| 0.4 | G1 | 0.38 | 0.38 | 0.39 | 0.38 | 0.38 |
| 0.4 | G2 | 0.38 | 0.37 | 0.39 | 0.37 | 0.39 |
| 0.4 | G3 | 0.38 | 0.37 | 0.40 | 0.37 | 0.39 |
| 0.4 | G4 | 0.38 | 0.37 | 0.40 | 0.36 | 0.39 |
| 0.4 | G5 | 0.37 | 0.35 | 0.39 | 0.36 | 0.38 |
| 0.4 | G6 | 0.37 | 0.34 | 0.37 | 0.35 | 0.38 |
| 0.4 | G7 | 0.37 | 0.34 | 0.37 | 0.34 | 0.37 |
| 0.4 | G8 | 0.37 | 0.34 | 0.37 | 0.33 | 0.36 |
|  |  |  |  |  |  |  |
| 0.5 | G0 | 0.47 | 0.47 | 0.48 | 0.47 | 0.48 |
| 0.5 | G1 | 0.40 | 0.40 | 0.42 | 0.41 | 0.41 |
| 0.5 | G2 | 0.41 | 0.40 | 0.43 | 0.40 | 0.42 |
| 0.5 | G3 | 0.41 | 0.40 | 0.43 | 0.40 | 0.43 |
| 0.5 | G4 | 0.42 | 0.39 | 0.42 | 0.39 | 0.42 |
| 0.5 | G5 | 0.40 | 0.38 | 0.42 | 0.39 | 0.42 |
| 0.5 | G6 | 0.41 | 0.37 | 0.41 | 0.37 | 0.41 |
| 0.5 | G7 | 0.40 | 0.37 | 0.41 | 0.38 | 0.40 |
| 0.5 | G8 | 0.40 | 0.36 | 0.41 | 0.36 | 0.40 |
|  |  |  |  |  |  |  |
| 0.6 | G0 | 0.49 | 0.48 | 0.50 | 0.48 | 0.49 |
| 0.6 | G1 | 0.43 | 0.42 | 0.44 | 0.43 | 0.43 |
| 0.6 | G2 | 0.44 | 0.42 | 0.46 | 0.43 | 0.45 |
| 0.6 | G3 | 0.44 | 0.42 | 0.46 | 0.43 | 0.46 |
| 0.6 | G4 | 0.44 | 0.41 | 0.45 | 0.42 | 0.45 |
| 0.6 | G5 | 0.44 | 0.41 | 0.45 | 0.40 | 0.45 |
| 0.6 | G6 | 0.44 | 0.41 | 0.44 | 0.39 | 0.45 |
| 0.6 | G7 | 0.43 | 0.40 | 0.45 | 0.38 | 0.43 |
| 0.6 | G8 | 0.41 | 0.39 | 0.44 | 0.38 | 0.42 |
|  |  |  |  |  |  |  |
| 0.7 | G0 | 0.50 | 0.50 | 0.51 | 0.50 | 0.50 |
| 0.7 | G1 | 0.45 | 0.43 | 0.45 | 0.45 | 0.46 |
| 0.7 | G2 | 0.47 | 0.44 | 0.48 | 0.46 | 0.48 |
| 0.7 | G3 | 0.49 | 0.44 | 0.49 | 0.46 | 0.49 |
| 0.7 | G4 | 0.48 | 0.43 | 0.49 | 0.45 | 0.49 |
| 0.7 | G5 | 0.47 | 0.43 | 0.48 | 0.44 | 0.47 |
| 0.7 | G6 | 0.46 | 0.42 | 0.47 | 0.43 | 0.47 |
| 0.7 | G7 | 0.44 | 0.41 | 0.45 | 0.42 | 0.45 |
| 0.7 | G8 | 0.46 | 0.40 | 0.45 | 0.40 | 0.44 |
|  |  |  |  |  |  |  |
| 0.8 | G0 | 0.52 | 0.51 | 0.52 | 0.52 | 0.52 |
| 0.8 | G1 | 0.48 | 0.47 | 0.49 | 0.47 | 0.48 |
| 0.8 | G2 | 0.51 | 0.48 | 0.52 | 0.48 | 0.50 |
| 0.8 | G3 | 0.52 | 0.47 | 0.52 | 0.48 | 0.51 |
| 0.8 | G4 | 0.52 | 0.47 | 0.51 | 0.48 | 0.51 |
| 0.8 | G5 | 0.51 | 0.46 | 0.51 | 0.47 | 0.51 |
| 0.8 | G6 | 0.49 | 0.44 | 0.51 | 0.46 | 0.50 |
| 0.8 | G7 | 0.48 | 0.43 | 0.50 | 0.43 | 0.48 |
| 0.8 | G8 | 0.48 | 0.42 | 0.49 | 0.44 | 0.47 |

**Table S5 Efficiency of the ssGBLUP in terms of their cumulative genetic response and reliability for all ssGBLUP scenarios when genotyping criterion was the candidates’ own phenotypes**

| p | gen | Cumulated gain | | | Reliability | | |
| --- | --- | --- | --- | --- | --- | --- | --- |
|  |  | RANDOM | TOP | EXTREME | RANDOM | TOP | EXTREME |
| 0.1 | G0 | - | - | - | 0.172 | 0.185 | 0.191 |
| 0.1 | G1 | 0.201 | 0.313 | 0.253 | 0.074 | 0.091 | 0.083 |
| 0.1 | G2 | 0.149 | 0.232 | 0.160 | 0.054 | 0.087 | 0.085 |
| 0.1 | G3 | 0.146 | 0.249 | 0.162 | 0.091 | 0.082 | 0.092 |
| 0.1 | G4 | 0.161 | 0.249 | 0.160 | 0.084 | 0.076 | 0.110 |
| 0.1 | G5 | 0.156 | 0.247 | 0.162 | 0.082 | 0.088 | 0.098 |
| 0.1 | G6 | 0.144 | 0.249 | 0.156 | 0.086 | 0.078 | 0.095 |
| 0.1 | G7 | 0.133 | 0.257 | 0.158 | 0.097 | 0.111 | 0.117 |
| 0.1 | G8 | 0.141 | 0.271 | 0.167 | 0.078 | 0.068 | 0.101 |
| 0.1 | mean | 0.154 | 0.258 | 0.172 | 0.091 | 0.096 | 0.108 |
| 0.2 | G0 | - | - | - | 0.246 | 0.259 | 0.273 |
| 0.2 | G1 | 0.300 | 0.430 | 0.329 | 0.143 | 0.182 | 0.192 |
| 0.2 | G2 | 0.226 | 0.420 | 0.296 | 0.150 | 0.163 | 0.188 |
| 0.2 | G3 | 0.235 | 0.422 | 0.301 | 0.187 | 0.172 | 0.195 |
| 0.2 | G4 | 0.228 | 0.438 | 0.311 | 0.160 | 0.151 | 0.217 |
| 0.2 | G5 | 0.227 | 0.429 | 0.315 | 0.162 | 0.144 | 0.213 |
| 0.2 | G6 | 0.223 | 0.421 | 0.322 | 0.168 | 0.150 | 0.201 |
| 0.2 | G7 | 0.212 | 0.411 | 0.326 | 0.173 | 0.139 | 0.209 |
| 0.2 | G8 | 0.205 | 0.402 | 0.331 | 0.167 | 0.129 | 0.201 |
| 0.2 | mean | 0.232 | 0.422 | 0.317 | 0.173 | 0.165 | 0.210 |
| 0.3 | G0 | - | - | - | 0.312 | 0.327 | 0.358 |
| 0.3 | G1 | 0.373 | 0.538 | 0.412 | 0.247 | 0.264 | 0.293 |
| 0.3 | G2 | 0.348 | 0.495 | 0.375 | 0.252 | 0.228 | 0.254 |
| 0.3 | G3 | 0.374 | 0.486 | 0.356 | 0.249 | 0.242 | 0.278 |
| 0.3 | G4 | 0.363 | 0.509 | 0.372 | 0.266 | 0.247 | 0.264 |
| 0.3 | G5 | 0.362 | 0.525 | 0.369 | 0.269 | 0.214 | 0.293 |
| 0.3 | G6 | 0.365 | 0.514 | 0.370 | 0.276 | 0.217 | 0.280 |
| 0.3 | G7 | 0.370 | 0.517 | 0.368 | 0.267 | 0.244 | 0.280 |
| 0.3 | G8 | 0.364 | 0.526 | 0.369 | 0.238 | 0.227 | 0.266 |
| 0.3 | mean | 0.365 | 0.514 | 0.374 | 0.264 | 0.246 | 0.285 |
| 0.4 | G0 | - | - | - | 0.395 | 0.387 | 0.439 |
| 0.4 | G1 | 0.423 | 0.629 | 0.492 | 0.347 | 0.341 | 0.397 |
| 0.4 | G2 | 0.417 | 0.606 | 0.477 | 0.318 | 0.306 | 0.348 |
| 0.4 | G3 | 0.423 | 0.601 | 0.486 | 0.340 | 0.316 | 0.384 |
| 0.4 | G4 | 0.425 | 0.623 | 0.490 | 0.333 | 0.308 | 0.388 |
| 0.4 | G5 | 0.422 | 0.636 | 0.508 | 0.346 | 0.282 | 0.384 |
| 0.4 | G6 | 0.432 | 0.625 | 0.509 | 0.357 | 0.265 | 0.356 |
| 0.4 | G7 | 0.436 | 0.611 | 0.503 | 0.359 | 0.291 | 0.364 |
| 0.4 | G8 | 0.446 | 0.610 | 0.517 | 0.353 | 0.277 | 0.361 |
| 0.4 | mean | 0.428 | 0.618 | 0.498 | 0.350 | 0.308 | 0.380 |
| 0.5 | G0 | - | - | - | 0.471 | 0.456 | 0.513 |
| 0.5 | G1 | 0.530 | 0.677 | 0.567 | 0.429 | 0.417 | 0.497 |
| 0.5 | G2 | 0.494 | 0.666 | 0.584 | 0.421 | 0.375 | 0.477 |
| 0.5 | G3 | 0.517 | 0.651 | 0.594 | 0.430 | 0.389 | 0.482 |
| 0.5 | G4 | 0.515 | 0.676 | 0.615 | 0.448 | 0.365 | 0.456 |
| 0.5 | G5 | 0.521 | 0.684 | 0.621 | 0.414 | 0.361 | 0.466 |
| 0.5 | G6 | 0.514 | 0.684 | 0.622 | 0.453 | 0.361 | 0.455 |
| 0.5 | G7 | 0.519 | 0.688 | 0.625 | 0.424 | 0.350 | 0.452 |
| 0.5 | G8 | 0.520 | 0.694 | 0.630 | 0.415 | 0.337 | 0.443 |
| 0.5 | mean | 0.516 | 0.677 | 0.607 | 0.434 | 0.379 | 0.471 |
| 0.6 | G0 | - | - | - | 0.555 | 0.524 | 0.598 |
| 0.6 | G1 | 0.591 | 0.732 | 0.683 | 0.535 | 0.491 | 0.564 |
| 0.6 | G2 | 0.611 | 0.752 | 0.685 | 0.527 | 0.454 | 0.568 |
| 0.6 | G3 | 0.629 | 0.749 | 0.677 | 0.503 | 0.464 | 0.578 |
| 0.6 | G4 | 0.639 | 0.758 | 0.699 | 0.517 | 0.426 | 0.548 |
| 0.6 | G5 | 0.649 | 0.765 | 0.702 | 0.531 | 0.452 | 0.548 |
| 0.6 | G6 | 0.655 | 0.766 | 0.695 | 0.531 | 0.462 | 0.535 |
| 0.6 | G7 | 0.668 | 0.780 | 0.699 | 0.505 | 0.428 | 0.545 |
| 0.6 | G8 | 0.662 | 0.786 | 0.703 | 0.446 | 0.406 | 0.513 |
| 0.6 | mean | 0.638 | 0.761 | 0.693 | 0.517 | 0.456 | 0.555 |
| 0.7 | G0 | - | - | - | 0.630 | 0.598 | 0.675 |
| 0.7 | G1 | 0.664 | 0.768 | 0.768 | 0.608 | 0.552 | 0.626 |
| 0.7 | G2 | 0.683 | 0.758 | 0.736 | 0.614 | 0.524 | 0.645 |
| 0.7 | G3 | 0.710 | 0.778 | 0.753 | 0.645 | 0.506 | 0.656 |
| 0.7 | G4 | 0.734 | 0.786 | 0.785 | 0.619 | 0.492 | 0.644 |
| 0.7 | G5 | 0.747 | 0.794 | 0.812 | 0.593 | 0.489 | 0.613 |
| 0.7 | G6 | 0.748 | 0.799 | 0.820 | 0.571 | 0.486 | 0.603 |
| 0.7 | G7 | 0.751 | 0.807 | 0.814 | 0.538 | 0.463 | 0.558 |
| 0.7 | G8 | 0.743 | 0.813 | 0.806 | 0.567 | 0.426 | 0.532 |
| 0.7 | mean | 0.722 | 0.788 | 0.787 | 0.598 | 0.504 | 0.617 |
| 0.8 | G0 | - | - | - | 0.725 | 0.680 | 0.748 |
| 0.8 | G1 | 0.794 | 0.844 | 0.833 | 0.726 | 0.673 | 0.761 |
| 0.8 | G2 | 0.788 | 0.848 | 0.834 | 0.725 | 0.634 | 0.747 |
| 0.8 | G3 | 0.800 | 0.858 | 0.841 | 0.734 | 0.605 | 0.743 |
| 0.8 | G4 | 0.835 | 0.859 | 0.842 | 0.722 | 0.596 | 0.711 |
| 0.8 | G5 | 0.852 | 0.886 | 0.841 | 0.713 | 0.564 | 0.688 |
| 0.8 | G6 | 0.861 | 0.885 | 0.849 | 0.662 | 0.528 | 0.707 |
| 0.8 | G7 | 0.859 | 0.884 | 0.861 | 0.633 | 0.503 | 0.680 |
| 0.8 | G8 | 0.848 | 0.880 | 0.876 | 0.616 | 0.473 | 0.636 |
| 0.8 | mean | 0.830 | 0.868 | 0.847 | 0.695 | 0.584 | 0.713 |

**Table S6 Efficiency of the ssGBLUP in terms of their cumulative genetic response and reliability for all ssGBLUP scenarios when genotyping criterion was the candidates’ estimated breeding values.**

| p | gen | Cumulated gain | | | Reliability | | |
| --- | --- | --- | --- | --- | --- | --- | --- |
|  |  | RANDOM | TOP | EXTREME | RANDOM | TOP | EXTREME |
| 0.1 | G0 | - | - | - | 0.172 | 0.181 | 0.177 |
| 0.1 | G1 | 0.201 | 0.296 | 0.185 | 0.074 | 0.096 | 0.096 |
| 0.1 | G2 | 0.149 | 0.262 | 0.130 | 0.054 | 0.108 | 0.096 |
| 0.1 | G3 | 0.146 | 0.291 | 0.139 | 0.091 | 0.097 | 0.102 |
| 0.1 | G4 | 0.161 | 0.300 | 0.149 | 0.084 | 0.086 | 0.108 |
| 0.1 | G5 | 0.156 | 0.297 | 0.163 | 0.082 | 0.056 | 0.120 |
| 0.1 | G6 | 0.144 | 0.285 | 0.154 | 0.086 | 0.070 | 0.104 |
| 0.1 | G7 | 0.133 | 0.275 | 0.150 | 0.097 | 0.081 | 0.118 |
| 0.1 | G8 | 0.141 | 0.275 | 0.158 | 0.078 | 0.055 | 0.105 |
| 0.1 | mean | 0.154 | 0.285 | 0.153 | 0.091 | 0.092 | 0.114 |
| 0.2 | G0 | - | - | - | 0.246 | 0.252 | 0.257 |
| 0.2 | G1 | 0.300 | 0.493 | 0.289 | 0.143 | 0.162 | 0.194 |
| 0.2 | G2 | 0.226 | 0.468 | 0.237 | 0.150 | 0.149 | 0.181 |
| 0.2 | G3 | 0.235 | 0.453 | 0.242 | 0.187 | 0.136 | 0.186 |
| 0.2 | G4 | 0.228 | 0.443 | 0.246 | 0.160 | 0.136 | 0.208 |
| 0.2 | G5 | 0.227 | 0.438 | 0.265 | 0.162 | 0.146 | 0.220 |
| 0.2 | G6 | 0.223 | 0.443 | 0.284 | 0.168 | 0.126 | 0.209 |
| 0.2 | G7 | 0.212 | 0.448 | 0.285 | 0.173 | 0.154 | 0.225 |
| 0.2 | G8 | 0.205 | 0.458 | 0.294 | 0.167 | 0.133 | 0.172 |
| 0.2 | mean | 0.232 | 0.456 | 0.268 | 0.173 | 0.155 | 0.206 |
| 0.3 | G0 | - | - | - | 0.312 | 0.319 | 0.330 |
| 0.3 | G1 | 0.373 | 0.604 | 0.428 | 0.247 | 0.270 | 0.302 |
| 0.3 | G2 | 0.348 | 0.592 | 0.431 | 0.252 | 0.238 | 0.271 |
| 0.3 | G3 | 0.374 | 0.604 | 0.418 | 0.249 | 0.221 | 0.265 |
| 0.3 | G4 | 0.363 | 0.611 | 0.432 | 0.266 | 0.220 | 0.282 |
| 0.3 | G5 | 0.362 | 0.615 | 0.434 | 0.269 | 0.210 | 0.298 |
| 0.3 | G6 | 0.365 | 0.605 | 0.442 | 0.276 | 0.208 | 0.277 |
| 0.3 | G7 | 0.370 | 0.601 | 0.445 | 0.267 | 0.198 | 0.289 |
| 0.3 | G8 | 0.364 | 0.596 | 0.450 | 0.238 | 0.184 | 0.261 |
| 0.3 | mean | 0.365 | 0.603 | 0.435 | 0.264 | 0.230 | 0.286 |
| 0.4 | G0 | - | - | - | 0.395 | 0.389 | 0.408 |
| 0.4 | G1 | 0.423 | 0.691 | 0.493 | 0.347 | 0.347 | 0.352 |
| 0.4 | G2 | 0.417 | 0.685 | 0.491 | 0.318 | 0.311 | 0.374 |
| 0.4 | G3 | 0.423 | 0.661 | 0.520 | 0.340 | 0.293 | 0.364 |
| 0.4 | G4 | 0.425 | 0.656 | 0.532 | 0.333 | 0.278 | 0.376 |
| 0.4 | G5 | 0.422 | 0.650 | 0.546 | 0.346 | 0.304 | 0.371 |
| 0.4 | G6 | 0.432 | 0.659 | 0.540 | 0.357 | 0.296 | 0.380 |
| 0.4 | G7 | 0.436 | 0.674 | 0.545 | 0.359 | 0.287 | 0.372 |
| 0.4 | G8 | 0.446 | 0.677 | 0.543 | 0.353 | 0.267 | 0.340 |
| 0.4 | mean | 0.428 | 0.669 | 0.526 | 0.350 | 0.308 | 0.371 |
| 0.5 | G0 | - | - | - | 0.471 | 0.460 | 0.487 |
| 0.5 | G1 | 0.530 | 0.741 | 0.593 | 0.429 | 0.448 | 0.480 |
| 0.5 | G2 | 0.494 | 0.762 | 0.617 | 0.421 | 0.391 | 0.453 |
| 0.5 | G3 | 0.517 | 0.753 | 0.630 | 0.430 | 0.386 | 0.470 |
| 0.5 | G4 | 0.515 | 0.761 | 0.638 | 0.448 | 0.378 | 0.445 |
| 0.5 | G5 | 0.521 | 0.776 | 0.638 | 0.414 | 0.375 | 0.465 |
| 0.5 | G6 | 0.514 | 0.778 | 0.640 | 0.453 | 0.349 | 0.462 |
| 0.5 | G7 | 0.519 | 0.782 | 0.644 | 0.424 | 0.379 | 0.431 |
| 0.5 | G8 | 0.520 | 0.795 | 0.639 | 0.415 | 0.336 | 0.416 |
| 0.5 | mean | 0.516 | 0.768 | 0.630 | 0.434 | 0.389 | 0.457 |
| 0.6 | G0 | - | - | - | 0.555 | 0.539 | 0.567 |
| 0.6 | G1 | 0.591 | 0.825 | 0.661 | 0.535 | 0.526 | 0.543 |
| 0.6 | G2 | 0.611 | 0.839 | 0.657 | 0.527 | 0.486 | 0.532 |
| 0.6 | G3 | 0.629 | 0.825 | 0.670 | 0.503 | 0.476 | 0.557 |
| 0.6 | G4 | 0.639 | 0.843 | 0.711 | 0.517 | 0.449 | 0.542 |
| 0.6 | G5 | 0.649 | 0.836 | 0.714 | 0.531 | 0.424 | 0.545 |
| 0.6 | G6 | 0.655 | 0.829 | 0.719 | 0.531 | 0.395 | 0.541 |
| 0.6 | G7 | 0.668 | 0.826 | 0.717 | 0.505 | 0.383 | 0.502 |
| 0.6 | G8 | 0.662 | 0.818 | 0.711 | 0.446 | 0.368 | 0.475 |
| 0.6 | mean | 0.638 | 0.830 | 0.695 | 0.517 | 0.450 | 0.534 |
| 0.7 | G0 | - | - | - | 0.630 | 0.621 | 0.644 |
| 0.7 | G1 | 0.664 | 0.862 | 0.763 | 0.608 | 0.598 | 0.642 |
| 0.7 | G2 | 0.683 | 0.888 | 0.749 | 0.614 | 0.577 | 0.625 |
| 0.7 | G3 | 0.710 | 0.884 | 0.745 | 0.645 | 0.556 | 0.645 |
| 0.7 | G4 | 0.734 | 0.882 | 0.782 | 0.619 | 0.525 | 0.634 |
| 0.7 | G5 | 0.747 | 0.875 | 0.791 | 0.593 | 0.521 | 0.608 |
| 0.7 | G6 | 0.748 | 0.878 | 0.794 | 0.571 | 0.499 | 0.599 |
| 0.7 | G7 | 0.751 | 0.870 | 0.793 | 0.538 | 0.484 | 0.553 |
| 0.7 | G8 | 0.743 | 0.868 | 0.783 | 0.567 | 0.430 | 0.520 |
| 0.7 | mean | 0.722 | 0.876 | 0.775 | 0.598 | 0.535 | 0.608 |
| 0.8 | G0 | - | - | - | 0.725 | 0.713 | 0.737 |
| 0.8 | G1 | 0.794 | 0.905 | 0.827 | 0.726 | 0.688 | 0.737 |
| 0.8 | G2 | 0.788 | 0.903 | 0.843 | 0.725 | 0.633 | 0.702 |
| 0.8 | G3 | 0.800 | 0.890 | 0.838 | 0.734 | 0.630 | 0.716 |
| 0.8 | G4 | 0.835 | 0.886 | 0.845 | 0.722 | 0.609 | 0.695 |
| 0.8 | G5 | 0.852 | 0.890 | 0.854 | 0.713 | 0.584 | 0.710 |
| 0.8 | G6 | 0.861 | 0.889 | 0.861 | 0.662 | 0.582 | 0.670 |
| 0.8 | G7 | 0.859 | 0.897 | 0.869 | 0.633 | 0.508 | 0.633 |
| 0.8 | G8 | 0.848 | 0.887 | 0.857 | 0.616 | 0.516 | 0.582 |
| 0.8 | mean | 0.830 | 0.893 | 0.849 | 0.695 | 0.607 | 0.687 |

**Table S7. GEBV Reliability estimates observed for all ssGBLUP scenarios**

| P | gen | Genotyped Candidates | | | | | Non Genotyped Candidates | | | | |
| --- | --- | --- | --- | --- | --- | --- | --- | --- | --- | --- | --- |
|  |  | RANDOM | TOP (PHE) | EXTREME (PHE) | TOP (EBV) | EXTREME (EBV) | RANDOM | TOP (PHE) | EXTREME (PHE) | TOP (EBV) | EXTREME (EBV) |
| 0.1 | G0 | 0.528 | 0.431 | 0.714 | 0.303 | 0.796 | 0.404 | 0.373 | 0.362 | 0.336 | 0.309 |
| 0.1 | G1 | 0.461 | 0.419 | 0.676 | 0.362 | 0.735 | 0.288 | 0.253 | 0.241 | 0.227 | 0.211 |
| 0.1 | G2 | 0.481 | 0.442 | 0.700 | 0.405 | 0.742 | 0.269 | 0.244 | 0.231 | 0.226 | 0.206 |
| 0.1 | G3 | 0.524 | 0.462 | 0.713 | 0.432 | 0.767 | 0.273 | 0.237 | 0.226 | 0.221 | 0.195 |
| 0.1 | G4 | 0.535 | 0.477 | 0.723 | 0.436 | 0.753 | 0.256 | 0.227 | 0.220 | 0.211 | 0.195 |
| 0.1 | G5 | 0.551 | 0.496 | 0.724 | 0.453 | 0.763 | 0.243 | 0.217 | 0.205 | 0.190 | 0.186 |
| 0.1 | G6 | 0.551 | 0.513 | 0.722 | 0.474 | 0.755 | 0.237 | 0.207 | 0.197 | 0.187 | 0.175 |
| 0.1 | G7 | 0.563 | 0.559 | 0.723 | 0.494 | 0.771 | 0.229 | 0.209 | 0.198 | 0.180 | 0.166 |
| 0.1 | G8 | 0.589 | 0.561 | 0.720 | 0.510 | 0.759 | 0.216 | 0.190 | 0.191 | 0.167 | 0.165 |
|  |  |  |  |  |  |  |  |  |  |  |  |
| 0.2 | G0 | 0.528 | 0.446 | 0.681 | 0.325 | 0.750 | 0.405 | 0.357 | 0.337 | 0.298 | 0.249 |
| 0.2 | G1 | 0.479 | 0.440 | 0.649 | 0.376 | 0.699 | 0.286 | 0.238 | 0.221 | 0.196 | 0.165 |
| 0.2 | G2 | 0.506 | 0.467 | 0.675 | 0.415 | 0.708 | 0.278 | 0.227 | 0.208 | 0.192 | 0.161 |
| 0.2 | G3 | 0.543 | 0.490 | 0.690 | 0.445 | 0.720 | 0.276 | 0.226 | 0.203 | 0.186 | 0.154 |
| 0.2 | G4 | 0.571 | 0.508 | 0.703 | 0.473 | 0.722 | 0.249 | 0.210 | 0.202 | 0.176 | 0.159 |
| 0.2 | G5 | 0.573 | 0.511 | 0.700 | 0.481 | 0.734 | 0.237 | 0.197 | 0.193 | 0.167 | 0.155 |
| 0.2 | G6 | 0.585 | 0.536 | 0.688 | 0.495 | 0.732 | 0.229 | 0.191 | 0.190 | 0.158 | 0.143 |
| 0.2 | G7 | 0.587 | 0.550 | 0.701 | 0.519 | 0.730 | 0.221 | 0.177 | 0.175 | 0.155 | 0.144 |
| 0.2 | G8 | 0.598 | 0.552 | 0.701 | 0.531 | 0.719 | 0.216 | 0.172 | 0.179 | 0.150 | 0.129 |
|  |  |  |  |  |  |  |  |  |  |  |  |
| 0.3 | G0 | 0.522 | 0.451 | 0.653 | 0.340 | 0.714 | 0.409 | 0.344 | 0.320 | 0.265 | 0.198 |
| 0.3 | G1 | 0.498 | 0.442 | 0.622 | 0.395 | 0.665 | 0.289 | 0.225 | 0.210 | 0.181 | 0.138 |
| 0.3 | G2 | 0.540 | 0.473 | 0.647 | 0.433 | 0.682 | 0.277 | 0.211 | 0.183 | 0.180 | 0.132 |
| 0.3 | G3 | 0.562 | 0.501 | 0.668 | 0.465 | 0.683 | 0.260 | 0.212 | 0.181 | 0.166 | 0.130 |
| 0.3 | G4 | 0.582 | 0.526 | 0.669 | 0.487 | 0.698 | 0.252 | 0.201 | 0.167 | 0.159 | 0.128 |
| 0.3 | G5 | 0.581 | 0.518 | 0.673 | 0.503 | 0.706 | 0.243 | 0.185 | 0.173 | 0.146 | 0.126 |
| 0.3 | G6 | 0.601 | 0.533 | 0.668 | 0.506 | 0.703 | 0.233 | 0.174 | 0.167 | 0.143 | 0.118 |
| 0.3 | G7 | 0.595 | 0.560 | 0.674 | 0.524 | 0.706 | 0.222 | 0.172 | 0.156 | 0.128 | 0.115 |
| 0.3 | G8 | 0.591 | 0.568 | 0.675 | 0.528 | 0.699 | 0.211 | 0.168 | 0.154 | 0.121 | 0.109 |
|  |  |  |  |  |  |  |  |  |  |  |  |
| 0.4 | G0 | 0.532 | 0.457 | 0.633 | 0.361 | 0.683 | 0.409 | 0.333 | 0.304 | 0.240 | 0.158 |
| 0.4 | G1 | 0.510 | 0.446 | 0.608 | 0.407 | 0.625 | 0.291 | 0.215 | 0.193 | 0.162 | 0.113 |
| 0.4 | G2 | 0.534 | 0.474 | 0.630 | 0.450 | 0.660 | 0.273 | 0.203 | 0.171 | 0.159 | 0.117 |
| 0.4 | G3 | 0.561 | 0.509 | 0.651 | 0.466 | 0.669 | 0.262 | 0.197 | 0.174 | 0.157 | 0.111 |
| 0.4 | G4 | 0.570 | 0.524 | 0.666 | 0.481 | 0.683 | 0.248 | 0.184 | 0.164 | 0.142 | 0.109 |
| 0.4 | G5 | 0.586 | 0.523 | 0.659 | 0.512 | 0.679 | 0.235 | 0.173 | 0.161 | 0.139 | 0.106 |
| 0.4 | G6 | 0.597 | 0.525 | 0.647 | 0.522 | 0.680 | 0.229 | 0.158 | 0.154 | 0.128 | 0.108 |
| 0.4 | G7 | 0.602 | 0.547 | 0.647 | 0.533 | 0.683 | 0.223 | 0.158 | 0.153 | 0.118 | 0.102 |
| 0.4 | G8 | 0.610 | 0.545 | 0.646 | 0.522 | 0.675 | 0.215 | 0.155 | 0.159 | 0.117 | 0.093 |
|  |  |  |  |  |  |  |  |  |  |  |  |
| 0.5 | G0 | 0.538 | 0.467 | 0.615 | 0.382 | 0.658 | 0.407 | 0.322 | 0.291 | 0.218 | 0.122 |
| 0.5 | G1 | 0.508 | 0.444 | 0.597 | 0.425 | 0.615 | 0.293 | 0.207 | 0.181 | 0.151 | 0.094 |
| 0.5 | G2 | 0.540 | 0.479 | 0.627 | 0.462 | 0.639 | 0.281 | 0.183 | 0.173 | 0.149 | 0.094 |
| 0.5 | G3 | 0.569 | 0.508 | 0.640 | 0.491 | 0.653 | 0.257 | 0.183 | 0.164 | 0.135 | 0.096 |
| 0.5 | G4 | 0.593 | 0.518 | 0.637 | 0.503 | 0.649 | 0.245 | 0.170 | 0.153 | 0.131 | 0.097 |
| 0.5 | G5 | 0.579 | 0.527 | 0.641 | 0.519 | 0.659 | 0.226 | 0.158 | 0.153 | 0.125 | 0.095 |
| 0.5 | G6 | 0.601 | 0.535 | 0.633 | 0.512 | 0.659 | 0.226 | 0.156 | 0.147 | 0.110 | 0.092 |
| 0.5 | G7 | 0.592 | 0.546 | 0.640 | 0.535 | 0.648 | 0.210 | 0.141 | 0.140 | 0.117 | 0.086 |
| 0.5 | G8 | 0.606 | 0.545 | 0.633 | 0.523 | 0.648 | 0.201 | 0.142 | 0.145 | 0.112 | 0.083 |
|  |  |  |  |  |  |  |  |  |  |  |  |
| 0.6 | G0 | 0.542 | 0.474 | 0.600 | 0.405 | 0.632 | 0.409 | 0.311 | 0.285 | 0.198 | 0.092 |
| 0.6 | G1 | 0.515 | 0.452 | 0.577 | 0.435 | 0.588 | 0.303 | 0.195 | 0.167 | 0.133 | 0.081 |
| 0.6 | G2 | 0.559 | 0.488 | 0.614 | 0.487 | 0.614 | 0.275 | 0.176 | 0.157 | 0.134 | 0.084 |
| 0.6 | G3 | 0.563 | 0.512 | 0.629 | 0.501 | 0.634 | 0.249 | 0.169 | 0.152 | 0.131 | 0.095 |
| 0.6 | G4 | 0.577 | 0.518 | 0.625 | 0.503 | 0.641 | 0.245 | 0.155 | 0.148 | 0.122 | 0.087 |
| 0.6 | G5 | 0.590 | 0.532 | 0.624 | 0.498 | 0.641 | 0.234 | 0.154 | 0.141 | 0.114 | 0.085 |
| 0.6 | G6 | 0.595 | 0.545 | 0.623 | 0.495 | 0.638 | 0.220 | 0.155 | 0.134 | 0.101 | 0.084 |
| 0.6 | G7 | 0.589 | 0.537 | 0.626 | 0.496 | 0.622 | 0.200 | 0.140 | 0.141 | 0.098 | 0.078 |
| 0.6 | G8 | 0.567 | 0.539 | 0.617 | 0.499 | 0.613 | 0.185 | 0.137 | 0.138 | 0.101 | 0.075 |
|  |  |  |  |  |  |  |  |  |  |  |  |
| 0.7 | G0 | 0.544 | 0.484 | 0.587 | 0.427 | 0.608 | 0.406 | 0.298 | 0.275 | 0.180 | 0.072 |
| 0.7 | G1 | 0.519 | 0.457 | 0.555 | 0.453 | 0.573 | 0.286 | 0.185 | 0.158 | 0.118 | 0.074 |
| 0.7 | G2 | 0.560 | 0.492 | 0.601 | 0.499 | 0.600 | 0.267 | 0.164 | 0.143 | 0.120 | 0.076 |
| 0.7 | G3 | 0.584 | 0.501 | 0.612 | 0.504 | 0.617 | 0.259 | 0.153 | 0.144 | 0.126 | 0.077 |
| 0.7 | G4 | 0.581 | 0.511 | 0.617 | 0.509 | 0.622 | 0.248 | 0.152 | 0.144 | 0.110 | 0.079 |
| 0.7 | G5 | 0.576 | 0.523 | 0.604 | 0.515 | 0.610 | 0.226 | 0.136 | 0.134 | 0.108 | 0.083 |
| 0.7 | G6 | 0.571 | 0.528 | 0.604 | 0.510 | 0.610 | 0.198 | 0.125 | 0.121 | 0.099 | 0.075 |
| 0.7 | G7 | 0.557 | 0.521 | 0.580 | 0.501 | 0.588 | 0.189 | 0.122 | 0.119 | 0.108 | 0.073 |
| 0.7 | G8 | 0.581 | 0.512 | 0.569 | 0.489 | 0.583 | 0.195 | 0.119 | 0.130 | 0.090 | 0.061 |
|  |  |  |  |  |  |  |  |  |  |  |  |
| 0.8 | G0 | 0.548 | 0.497 | 0.573 | 0.457 | 0.588 | 0.405 | 0.297 | 0.277 | 0.163 | 0.063 |
| 0.8 | G1 | 0.527 | 0.478 | 0.559 | 0.467 | 0.557 | 0.301 | 0.172 | 0.150 | 0.110 | 0.069 |
| 0.8 | G2 | 0.567 | 0.509 | 0.592 | 0.496 | 0.582 | 0.271 | 0.160 | 0.148 | 0.112 | 0.081 |
| 0.8 | G3 | 0.581 | 0.513 | 0.600 | 0.514 | 0.597 | 0.264 | 0.144 | 0.135 | 0.113 | 0.080 |
| 0.8 | G4 | 0.589 | 0.527 | 0.597 | 0.517 | 0.596 | 0.233 | 0.131 | 0.131 | 0.109 | 0.085 |
| 0.8 | G5 | 0.590 | 0.518 | 0.589 | 0.515 | 0.608 | 0.225 | 0.133 | 0.128 | 0.090 | 0.075 |
| 0.8 | G6 | 0.571 | 0.509 | 0.599 | 0.516 | 0.588 | 0.189 | 0.115 | 0.115 | 0.091 | 0.070 |
| 0.8 | G7 | 0.564 | 0.505 | 0.590 | 0.486 | 0.576 | 0.178 | 0.117 | 0.119 | 0.087 | 0.064 |
| 0.8 | G8 | 0.561 | 0.497 | 0.580 | 0.492 | 0.556 | 0.176 | 0.118 | 0.112 | 0.105 | 0.064 |

**Table S8. Proportion of genotyped candidates, which were selected as parents for all ssGBLUP scenarios**

| P | gen | Male Candidates | | | | | Female Candidates | | | | |
| --- | --- | --- | --- | --- | --- | --- | --- | --- | --- | --- | --- |
|  |  | RANDOM | TOP (PHE) | EXTREME (PHE) | TOP (EBV) | EXTREME (EBV) | RANDOM | TOP (PHE) | EXTREME (PHE) | TOP (EBV) | EXTREME (EBV) |
| 0.1 | G0 | 0.124 | 0.479 | 0.290 | 0.736 | 0.423 | 0.101 | 0.181 | 0.100 | 0.198 | 0.100 |
| 0.1 | G1 | 0.155 | 0.509 | 0.321 | 0.699 | 0.391 | 0.098 | 0.178 | 0.099 | 0.192 | 0.101 |
| 0.1 | G2 | 0.168 | 0.528 | 0.333 | 0.686 | 0.386 | 0.100 | 0.175 | 0.102 | 0.189 | 0.101 |
| 0.1 | G3 | 0.169 | 0.499 | 0.327 | 0.671 | 0.386 | 0.101 | 0.172 | 0.102 | 0.185 | 0.101 |
| 0.1 | G4 | 0.183 | 0.514 | 0.325 | 0.657 | 0.376 | 0.098 | 0.170 | 0.104 | 0.183 | 0.103 |
| 0.1 | G5 | 0.179 | 0.502 | 0.332 | 0.629 | 0.366 | 0.102 | 0.170 | 0.103 | 0.181 | 0.104 |
| 0.1 | G6 | 0.170 | 0.500 | 0.331 | 0.612 | 0.360 | 0.100 | 0.168 | 0.104 | 0.180 | 0.103 |
| 0.1 | G7 | 0.170 | 0.491 | 0.314 | 0.585 | 0.363 | 0.102 | 0.166 | 0.106 | 0.177 | 0.104 |
| 0.1 | G8 | - | - | - | - | - | - | - | - | - | - |
|  |  |  |  |  |  |  |  |  |  |  |  |
| 0.2 | G0 | 0.253 | 0.723 | 0.490 | 0.985 | 0.734 | 0.202 | 0.341 | 0.201 | 0.387 | 0.200 |
| 0.2 | G1 | 0.281 | 0.777 | 0.534 | 0.965 | 0.697 | 0.201 | 0.336 | 0.202 | 0.365 | 0.203 |
| 0.2 | G2 | 0.309 | 0.772 | 0.553 | 0.950 | 0.665 | 0.201 | 0.331 | 0.202 | 0.358 | 0.201 |
| 0.2 | G3 | 0.315 | 0.782 | 0.563 | 0.935 | 0.656 | 0.199 | 0.326 | 0.205 | 0.349 | 0.204 |
| 0.2 | G4 | 0.337 | 0.773 | 0.541 | 0.919 | 0.661 | 0.200 | 0.324 | 0.207 | 0.343 | 0.207 |
| 0.2 | G5 | 0.336 | 0.767 | 0.553 | 0.912 | 0.630 | 0.205 | 0.320 | 0.206 | 0.341 | 0.205 |
| 0.2 | G6 | 0.320 | 0.749 | 0.550 | 0.894 | 0.623 | 0.201 | 0.318 | 0.208 | 0.337 | 0.207 |
| 0.2 | G7 | 0.308 | 0.745 | 0.550 | 0.881 | 0.607 | 0.197 | 0.315 | 0.210 | 0.331 | 0.206 |
| 0.2 | G8 | - | - | - | - | - | - | - | - | - | - |
|  |  |  |  |  |  |  |  |  |  |  |  |
| 0.3 | G0 | 0.358 | 0.855 | 0.641 | 0.996 | 0.924 | 0.300 | 0.486 | 0.303 | 0.562 | 0.301 |
| 0.3 | G1 | 0.423 | 0.889 | 0.702 | 0.996 | 0.884 | 0.297 | 0.479 | 0.299 | 0.528 | 0.302 |
| 0.3 | G2 | 0.452 | 0.914 | 0.722 | 0.994 | 0.876 | 0.305 | 0.476 | 0.304 | 0.507 | 0.306 |
| 0.3 | G3 | 0.491 | 0.907 | 0.714 | 0.987 | 0.844 | 0.302 | 0.467 | 0.309 | 0.501 | 0.310 |
| 0.3 | G4 | 0.479 | 0.897 | 0.734 | 0.982 | 0.825 | 0.302 | 0.465 | 0.305 | 0.492 | 0.311 |
| 0.3 | G5 | 0.471 | 0.895 | 0.707 | 0.978 | 0.802 | 0.302 | 0.461 | 0.309 | 0.486 | 0.309 |
| 0.3 | G6 | 0.465 | 0.891 | 0.704 | 0.966 | 0.804 | 0.300 | 0.457 | 0.308 | 0.479 | 0.310 |
| 0.3 | G7 | 0.442 | 0.899 | 0.695 | 0.964 | 0.784 | 0.298 | 0.450 | 0.310 | 0.474 | 0.306 |
| 0.3 | G8 | - | - | - | - | - | - | - | - | - | - |
|  |  |  |  |  |  |  |  |  |  |  |  |
| 0.4 | G0 | 0.462 | 0.933 | 0.743 | 0.999 | 0.984 | 0.405 | 0.613 | 0.403 | 0.721 | 0.403 |
| 0.4 | G1 | 0.543 | 0.954 | 0.815 | 0.999 | 0.956 | 0.398 | 0.615 | 0.400 | 0.673 | 0.402 |
| 0.4 | G2 | 0.574 | 0.957 | 0.833 | 0.997 | 0.943 | 0.402 | 0.608 | 0.407 | 0.652 | 0.409 |
| 0.4 | G3 | 0.583 | 0.962 | 0.829 | 0.994 | 0.933 | 0.400 | 0.602 | 0.406 | 0.639 | 0.411 |
| 0.4 | G4 | 0.596 | 0.967 | 0.842 | 0.995 | 0.918 | 0.401 | 0.594 | 0.410 | 0.631 | 0.410 |
| 0.4 | G5 | 0.579 | 0.952 | 0.842 | 0.994 | 0.904 | 0.401 | 0.593 | 0.408 | 0.619 | 0.409 |
| 0.4 | G6 | 0.577 | 0.955 | 0.820 | 0.992 | 0.891 | 0.398 | 0.587 | 0.411 | 0.614 | 0.409 |
| 0.4 | G7 | 0.560 | 0.953 | 0.819 | 0.990 | 0.874 | 0.399 | 0.583 | 0.407 | 0.606 | 0.409 |
| 0.4 | G8 | - | - | - | - | - | - | - | - | - | - |
|  |  |  |  |  |  |  |  |  |  |  |  |
| 0.5 | G0 | 0.584 | 0.968 | 0.823 | 1.000 | 0.995 | 0.499 | 0.723 | 0.503 | 0.857 | 0.504 |
| 0.5 | G1 | 0.661 | 0.979 | 0.878 | 1.000 | 0.983 | 0.498 | 0.735 | 0.501 | 0.805 | 0.505 |
| 0.5 | G2 | 0.694 | 0.989 | 0.894 | 1.000 | 0.973 | 0.501 | 0.727 | 0.505 | 0.781 | 0.507 |
| 0.5 | G3 | 0.692 | 0.982 | 0.904 | 0.997 | 0.964 | 0.501 | 0.719 | 0.507 | 0.764 | 0.511 |
| 0.5 | G4 | 0.704 | 0.987 | 0.893 | 0.999 | 0.956 | 0.499 | 0.715 | 0.508 | 0.753 | 0.510 |
| 0.5 | G5 | 0.684 | 0.983 | 0.897 | 0.996 | 0.946 | 0.502 | 0.711 | 0.507 | 0.746 | 0.509 |
| 0.5 | G6 | 0.670 | 0.977 | 0.890 | 0.996 | 0.934 | 0.502 | 0.711 | 0.508 | 0.737 | 0.505 |
| 0.5 | G7 | 0.666 | 0.978 | 0.891 | 0.997 | 0.938 | 0.496 | 0.705 | 0.505 | 0.730 | 0.506 |
| 0.5 | G8 | - | - | - | - | - | - | - | - | - | - |
|  |  |  |  |  |  |  |  |  |  |  |  |
| 0.6 | G0 | 0.678 | 0.984 | 0.883 | 1.000 | 0.997 | 0.601 | 0.821 | 0.601 | 0.956 | 0.604 |
| 0.6 | G1 | 0.744 | 0.990 | 0.921 | 0.999 | 0.990 | 0.599 | 0.832 | 0.601 | 0.906 | 0.604 |
| 0.6 | G2 | 0.776 | 0.993 | 0.937 | 1.000 | 0.986 | 0.600 | 0.828 | 0.605 | 0.880 | 0.607 |
| 0.6 | G3 | 0.791 | 0.993 | 0.941 | 1.000 | 0.988 | 0.600 | 0.825 | 0.604 | 0.870 | 0.607 |
| 0.6 | G4 | 0.773 | 0.993 | 0.938 | 0.999 | 0.977 | 0.598 | 0.820 | 0.612 | 0.862 | 0.610 |
| 0.6 | G5 | 0.771 | 0.995 | 0.940 | 0.998 | 0.964 | 0.603 | 0.819 | 0.604 | 0.852 | 0.610 |
| 0.6 | G6 | 0.764 | 0.994 | 0.941 | 0.997 | 0.957 | 0.600 | 0.817 | 0.606 | 0.845 | 0.606 |
| 0.6 | G7 | 0.755 | 0.991 | 0.928 | 0.998 | 0.945 | 0.598 | 0.816 | 0.604 | 0.842 | 0.602 |
| 0.6 | G8 | - | - | - | - | - | - | - | - | - | - |
|  |  |  |  |  |  |  |  |  |  |  |  |
| 0.7 | G0 | 0.761 | 0.994 | 0.928 | 1.000 | 0.998 | 0.705 | 0.901 | 0.701 | 0.995 | 0.703 |
| 0.7 | G1 | 0.826 | 0.997 | 0.958 | 1.000 | 0.995 | 0.697 | 0.910 | 0.702 | 0.973 | 0.711 |
| 0.7 | G2 | 0.843 | 0.998 | 0.966 | 1.000 | 0.988 | 0.699 | 0.909 | 0.706 | 0.955 | 0.705 |
| 0.7 | G3 | 0.847 | 0.998 | 0.971 | 1.000 | 0.986 | 0.700 | 0.911 | 0.701 | 0.944 | 0.708 |
| 0.7 | G4 | 0.853 | 0.998 | 0.964 | 0.999 | 0.979 | 0.702 | 0.907 | 0.704 | 0.937 | 0.712 |
| 0.7 | G5 | 0.856 | 0.996 | 0.957 | 0.999 | 0.967 | 0.700 | 0.907 | 0.704 | 0.932 | 0.705 |
| 0.7 | G6 | 0.831 | 0.995 | 0.952 | 0.999 | 0.958 | 0.699 | 0.908 | 0.703 | 0.932 | 0.703 |
| 0.7 | G7 | 0.825 | 0.995 | 0.954 | 0.999 | 0.961 | 0.698 | 0.908 | 0.702 | 0.927 | 0.702 |
| 0.7 | G8 | - | - | - | - | - | - | - | - | - | - |
|  |  |  |  |  |  |  |  |  |  |  |  |
| 0.8 | G0 | 0.848 | 0.998 | 0.957 | 1.000 | 0.999 | 0.798 | 0.954 | 0.802 | 1.000 | 0.802 |
| 0.8 | G1 | 0.888 | 0.999 | 0.977 | 1.000 | 0.996 | 0.801 | 0.966 | 0.798 | 0.996 | 0.801 |
| 0.8 | G2 | 0.914 | 1.000 | 0.978 | 1.000 | 0.994 | 0.800 | 0.964 | 0.806 | 0.989 | 0.804 |
| 0.8 | G3 | 0.908 | 0.998 | 0.977 | 1.000 | 0.989 | 0.798 | 0.964 | 0.803 | 0.985 | 0.802 |
| 0.8 | G4 | 0.903 | 0.999 | 0.982 | 1.000 | 0.984 | 0.795 | 0.965 | 0.801 | 0.980 | 0.805 |
| 0.8 | G5 | 0.903 | 0.998 | 0.977 | 1.000 | 0.977 | 0.798 | 0.966 | 0.798 | 0.979 | 0.800 |
| 0.8 | G6 | 0.903 | 0.998 | 0.969 | 1.000 | 0.972 | 0.800 | 0.968 | 0.799 | 0.978 | 0.801 |
| 0.8 | G7 | 0.875 | 0.996 | 0.977 | 0.999 | 0.964 | 0.795 | 0.967 | 0.793 | 0.977 | 0.796 |
| 0.8 | G8 | - |  | - | - | - | - | - | - | - | - |
